# Alcohol control policies add to secular trends in all-cause mortality rates in young adults

**DOI:** 10.1038/s41598-021-94562-1

**Published:** 2021-07-23

**Authors:** Alexander Tran, Jakob Manthey, Shannon Lange, Huan Jiang, Mindaugas Štelemėkas, Vaida Liutkutė-Gumarov, Olga Meščeriakova-Veliulienė, Janina Petkevičienė, Ričardas Radišauskas, Tadas Telksnys, Jürgen Rehm

**Affiliations:** 1 Institute for Mental Health Policy Research, Centre for Addiction and Mental Health (CAMH), 33 Russell Street, Toronto, ON M5S 2S1 Canada; 2 Institute of Clinical Psychology and Psychotherapy, Technische Universität Dresden, Chemnitzer Str. 46, 01187 Dresden, Germany; 3 Department of Psychiatry and Psychotherapy, Center for Interdisciplinary Addiction Research (ZIS), University Medical Center Hamburg-Eppendorf (UKE), Martinistraße 52, 20246 Hamburg, Germany; 4 Department of Psychiatry, Medical Faculty, University of Leipzig, Semmelweisstraße 10, 04103 Leipzig, Germany; 5 Campbell Family Mental Health Research Institute, CAMH, 250 College Street, Toronto, ON M5T 1R8 Canada; 6 Department of Psychiatry, University of Toronto, 250 College Street, 8th Floor, Toronto, ON M5T 1R8 Canada; 7 Dalla Lana School of Public Health, University of Toronto, 155 College Street, 6th Floor, Toronto, ON M5T 3M7 Canada; 8 Institute of Medical Science (IMS), University of Toronto, Medical Sciences Building, 1 King’s College Circle, Room 2374, Toronto, ON M5S 1A8 Canada; 9 Department of International Health Projects, Institute for Leadership and Health Management, I.M. Sechenov First Moscow State Medical University, Trubetskaya str., 8, b. 2, Moscow, Russian Federation 119992; 10 Health Research Institute, Faculty of Public Health, Lithuanian University of Health Sciences, Tilžės 18, 44307 Kaunas, Lithuania; 11 Department of Preventive Medicine, Faculty of Public Health, Lithuanian University of Health Sciences, Tilžės 18, 44307 Kaunas, Lithuania; 12 Department of Health Management, Faculty of Public Health, Lithuanian University of Health Sciences, Tilžės 18, 44307 Kaunas, Lithuania; 13 Department of Environmental and Occupational Medicine, Faculty of Public Health, Lithuanian University of Health Sciences, Tilžės 18, 44307 Kaunas, Lithuania; 14 Institute of Cardiology, Lithuanian University of Health Sciences, Sukilėlių av. 15, 50162 Kaunas, Lithuania

**Keywords:** Disease prevention, Health policy, Public health, Epidemiology

## Abstract

Alcohol consumption is a major risk factor for premature mortality. Although alcohol control policies are known to impact all-cause mortality rates, the effect that policies have on specific age groups is an important area of research. This study investigates the effect of alcohol control policies implemented in 2009 and 2017 in Lithuania on all-cause mortality rates. All-cause mortality rates (deaths per 100,000 people) were obtained for 2001–2018 by 10-year age groups (20–29, 30–39, 40–49 years, etc.). All-cause mortality rates, independent of macro-level secular trends (e.g., economic trends) were examined. Following a joinpoint analysis to control for secular trends, an interrupted time series analysis showed that alcohol control policies had a significant effect on all-cause mortality rates (*p* = .018), with the most significant impact occurring among young adults (20–29 and 30–39 years of age). For these age groups, their mortality rate decreased during the 12 months following policy implementation (following the policy in 2009 for those 20–29 years of age, *p* = .0026, and following the policy in 2017 for those 30–39 years of age, *p* = .011). The results indicate that alcohol control policy can impact all-cause mortality rates, above and beyond secular trends, and that the impact is significant among young adults.

## Introduction

Alcohol use is one of the biggest risk factors for burden of disease and premature mortality^1^, and has been causally related to more than 200 three-digit International Classification of Diseases, 10th revision (ICD-10) categories^2^, ranging from non-communicable disease to both unintentional and intentional injury^3,4^. Alcohol can contribute to all-cause mortality not only by way of alcohol-attributable disease (e.g., alcoholic cirrhosis of liver), but also by being a risk factor for certain causes of death (e.g., hypertension and atherosclerosis, violent injury and death)^5^. In some European countries (e.g., the Russian Federation and other former Soviet Union countries), the trends in alcohol consumption mirror the trends of mortality, especially among young men^6–8^. Compared to neighboring countries, there is a disparity of 10–15% in life expectancy and a ten-fold higher mortality rate (deaths per 10,000 people) in Central and Eastern European countries^9,10^, which have been partially attributed to high levels of alcohol consumption^6–9^. Consequently, alcohol control policies aimed at reducing alcohol consumption and lowering population-level mortality rates have been implemented to a differing extent in almost all countries^11,12^. The World Health Organization (WHO) recommends a set of policies as “best buys”, which are both effective and cost-effective to implement with respect to reducing alcohol-related harm. These alcohol control policies (increase of excise taxes, restrictions on physical availability, and bans or comprehensive restrictions on advertising and marketing) have been linked to a reduction in all-cause mortality rates in the Russian Federation^13^.

Notably, the risk of alcohol consumption on mortality (and its responsiveness to control policies) varies across population subgroups^11^. Acute harm from alcohol-use related injuries is more prevalent among younger adults, meanwhile chronic health conditions (e.g., cirrhosis or cardiomyopathy) resulting from prolonged alcohol-use are more prevalent among middle-aged and older adults^14,15^. In younger age groups, alcohol consumption is more likely to be characterized by heavy episodic drinking (HED), which has high (40–45%) prevalence rates among young adolescents (15–24 years old^12^). In young and middle-aged adults (15–49 years old), alcohol consumption is also a leading risk factor for premature death, whereas other risk factors, such as smoking and high BMI cause greater loss of life among older adults^16–18^. In one study, traffic accidents had a greater proportion of victims with blood alcohol levels > 10 mg/dl for adults 34 years of age and younger, compared to other adult age groups (35–74 years old)^19^.

The effects of alcohol consumption on morbidity and mortality varies across age groups^7,8,20,21^, as does the impact of alcohol control policies on mortality^22,23^. More specifically, increased alcohol taxation and restrictions on hours of sale causes alcohol to become less affordable and less available, which should have a greater effect for younger adults, who have lower income and are prone to HED, as has been shown in classic studies^11,24,25^. Indeed, lower socioeconomic status and lower education individuals are more likely to die due to alcohol-related causes following reduced alcohol taxation^23,26^.

For older adults, alcohol control policies that increase price and reduce availability during later hours may not be as impactful^18,19^. In other cases of availability restrictions however, the effects should be observed population-wide^27,28^. Older adults, on average, have more economic means, a higher rate of mortality, are less likely to engage in HED or drive under the influence of alcohol, and are more likely to die from causes that are unrelated to acute alcohol use. Yet, recent research has found that price decreases (i.e., increased affordability) was related to increases in alcohol consumption and alcohol-related mortality only in older adults in Finland^22,23^. These researchers studied a case of a natural experiment (mortality rates before and after a dramatic price decrease) and concluded that reduced taxation affected adults aged 40–49 (males) and 50–59 (females) by increasing alcohol-related mortality, but decreasing all-cause mortality in those older than 69 years of age. A key difference between past work and the current study is how confounding variables and latent trends are accounted for. A similar methodology (interrupted time series analysis) is employed here, but a joinpoint analysis is used to control for underlying secular trends. Using this approach we aimed to determine whether there is a relationship between taxation as well as alcohol availability, on all-cause mortality across age groups in Lithuania. Important to note, younger age groups have a lower absolute all-cause mortality rate, and so the beneficial effects of alcohol control policy on mortality may be best represented by relative changes in mortality rate^20^.

Lithuania had periods of economic decline in the past 2 decades, which occurred at the onset of multiple alcohol policies and led to dramatic changes in mortality^29^. In addition to taxation, policies enforcing reduced hours of alcohol sales in off-premises settings were also introduced at the same time^30^. We expected, given the unique economic circumstances in Lithuania, along with the combination of policies introduced, that young adults would exhibit a greater relative decrease in their all-cause mortality rate following the introduction of alcohol control policies^31^.

Štelemėkas and colleagues^32^ showed that all-cause mortality, across the entire population, decreased following policies that resulted in higher taxation and reduced availability of alcohol. In their work, they used an interrupted time-series analysis to test a number of alcohol control policies that were selected by policy experts and were part of the “best buys” recommendations. They found that two policies introduced in 2009 and 2017, were associated with marked decreases in all-cause mortality rates in Lithuania. As discussed above, however, there were confounding factors that may have affected the causal interpretation of these findings. For instance, in 2008–2009, alcohol control policies in Lithuania (including increased taxation, reduced availability, tougher drink-driving legislation, and restricted marketing^30,33^) were enacted during a severe recession, which was characterized with high stress levels, emigration, high unemployment rates, and lower average income^34^. Meanwhile, factors related to other socio-economic events were less prevalent during 2016–2018, when further alcohol control policies were implemented in Lithuania because at the time the economy was relatively stable, and marked with temperate economic growth^33^. Furthermore, it is unclear whether these policies affected each age groups within the population equally.

In an effort to isolate the effects of alcohol control policies on all-cause mortality, in the present study we aim to control for secular trends (e.g., political and economic) in all-cause mortality rate using population-level mortality data from Lithuania. To address the challenge of determining causality in quasi-experimental designs and time series data, we employ a two-stage data analysis technique in which a base model (joinpoint analysis) containing the underlying secular trends is created. Building on this base model, and on the work of Štelemėkas and colleagues^32^, we aim to determine whether the effect of two specific sets of alcohol control policies implemented in Lithuania have an effect on all-cause mortality rates when secular trends are used as a covariate. One set of policies was implemented on the 1st of January 2009, including: (1) taxation (increase excise tax by 10–15%, removal of tax exemptions for small beer breweries) and (2) changes in the availability of alcohol (off-premise sales restricted at night, a ban on having opened alcohol beverages in cars). On the 1st of March 2017 another set of taxation policy changes (increase excise tax: 111–112% for wines and beer, 23% for ethyl alcohol) was introduced (see Štelemėkas et al.^32^ for more details on all alcohol policies introduced between 2001 and 2018 and Supplementary Table S1 for details on the two policy sets). Furthermore, we aim to explore the effect of these two sets of policies on mortality rates across different age groups. Our specific hypotheses are as follows:

1. For the adult population, alcohol control policies of increasing taxation and decreasing availability implemented in 2009 and 2017 resulted in a decrease in all-cause mortality rates when controlling for the effect of secular trends in Lithuania.
2. The effects are most pronounced in the age groups of 20–29 years and 30–39 years. We expect a greater relative decrease in the mortality rates (as opposed to absolute change in mortality rates) for these age groups following the alcohol control policies implemented in 2009 and 2017 in Lithuania.

## Results

**Figure 1:**
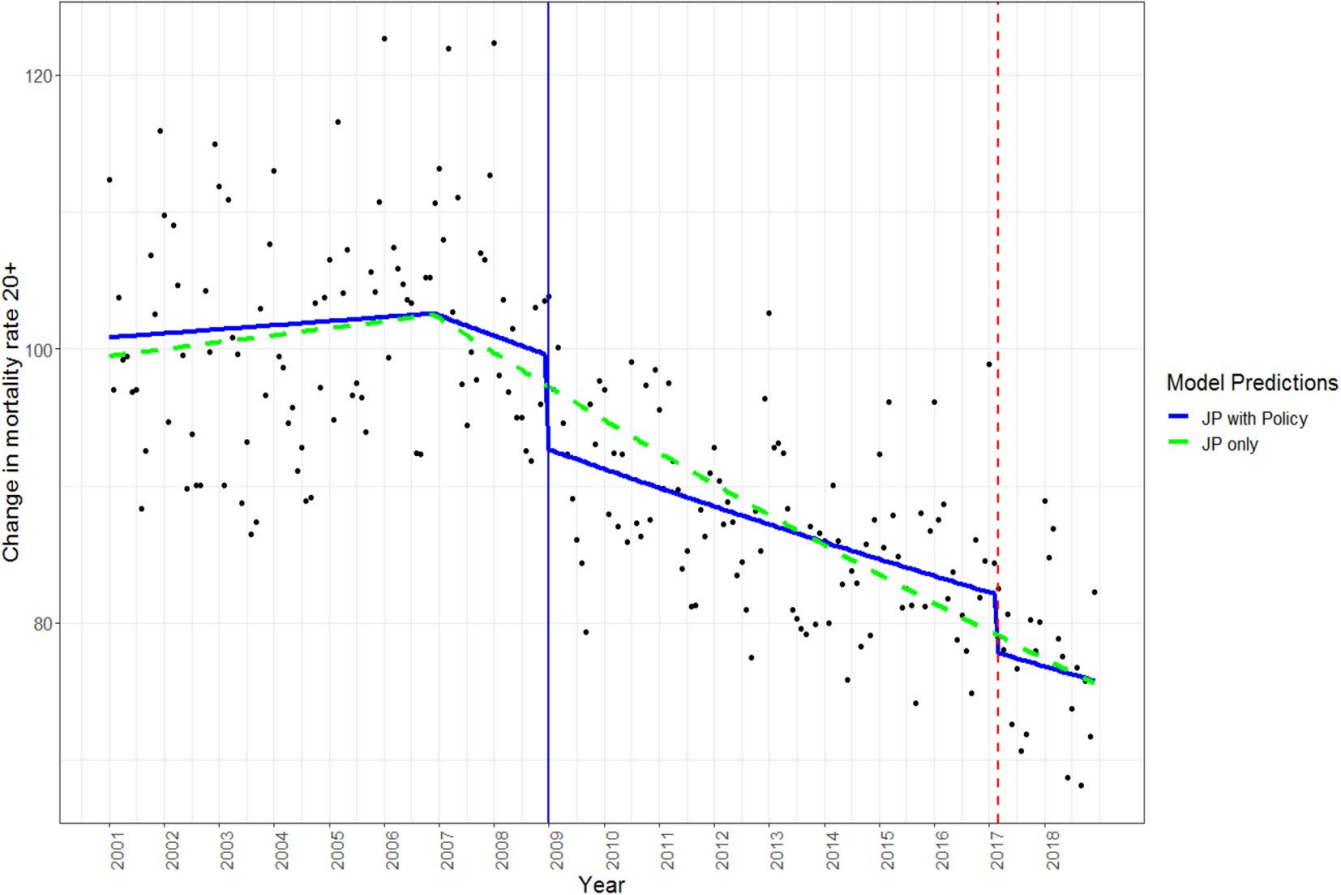
Scatterplot trend of mortality rate (deaths per 100,000 people) for all ages 20+. Joinpoint (JP) modeled with and without policy effects. Alcohol control policy implemented in 2009 (increased taxation and reduced availability) and 2017 (increased taxation) shown by the solid blue line and dashed red line, respectively.

**Figure 2:**
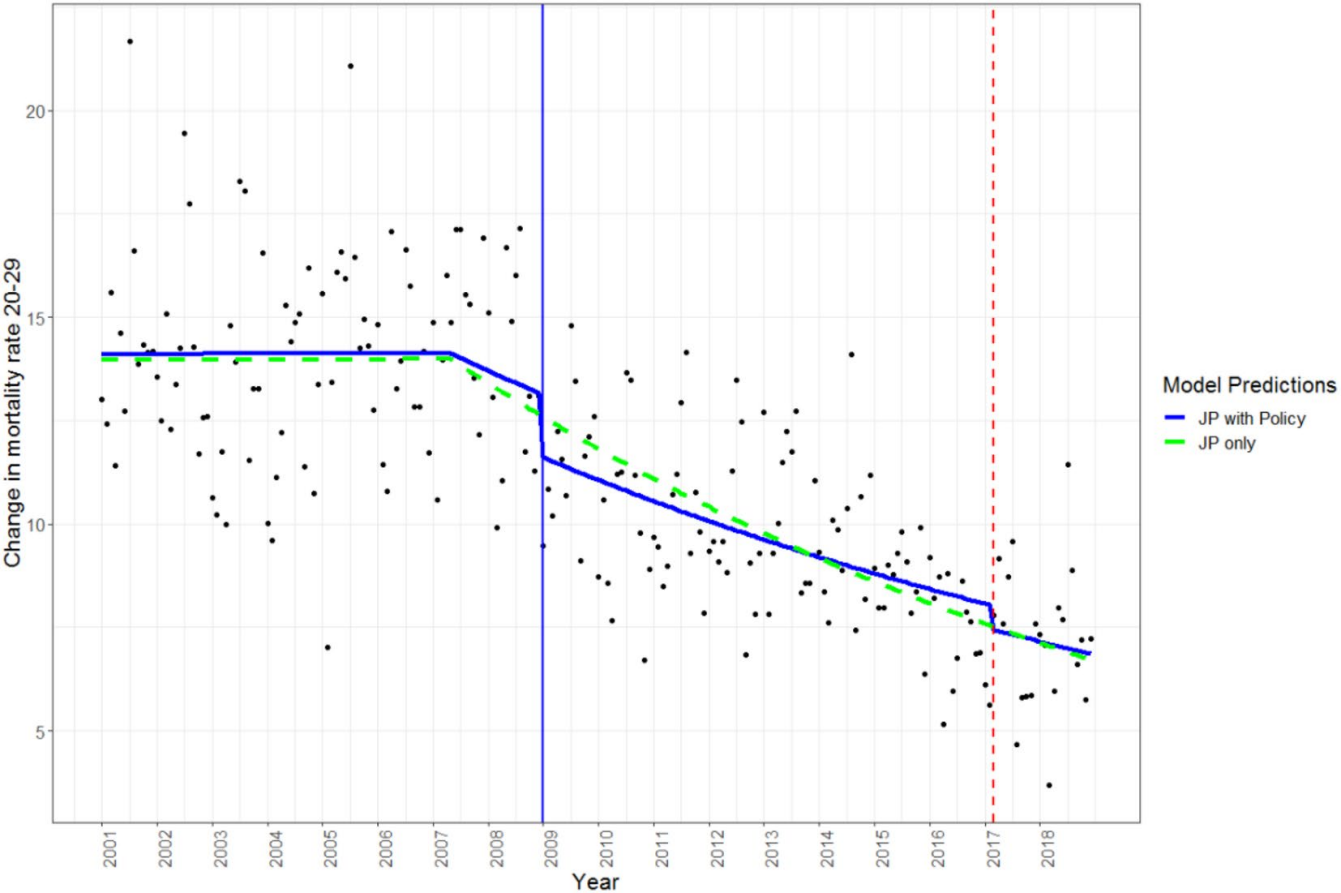
Scatterplot trend of mortality rate (deaths per 100,000 people) for ages 20–29. Joinpoint (JP) modeled with and without policy effects. Alcohol control policy implemented in 2009 (increased taxation and reduced availability) and 2017 (increased taxation) shown by the solid blue line and dashed red line, respectively.

The joinpoint analysis identified a similar secular trend across all age groups, with a slight variation in the number of joinpoints specified. Specifically, there were no joinpoints identified for ages 70–79 and 80+, one joinpoint for all ages 20+ (age-standardized), ages 20–29 and 60–69, two joinpoints for 50–59, and three joinpoints for 30–39 and 40–49 (see Table 1). Whenever a joinpoint was identified, there was a declining secular trend in the mortality rates following the joinpoint between January 2007 and January 2008. Figure 1 shows the secular trend for those 20+ years old and Fig. 2 shows the secular trend for those 20–29 years old (note that similar trends were observed in all other age groups; see Supplementary Figures SF3-SF9). Autocorrelation was observed in the dataset and was corrected for, using an autoregressive integrated moving average (ARIMA) model (see Supplementary Figures SF10–SF17 for autocorrelation function and partial autocorrelation function plots). The general additive mixed model (GAMM) showed significant variance explained in both the joinpoint models (R^2^-adjusted = 0.67–0.87), and the joinpoint model with policies included (R^2^-adjusted = 0.68–0.87 see Table 2; for exact model details see Supplementary Table S5). In support of Hypothesis 1, for the aggregated adult population (20+ years) there was a significant effect of alcohol policy in the model above and beyond the secular trends based on the omnibus likelihood ratio test (Table 2). Furthermore, the effect of alcohol policy significantly reduced all-cause mortality for both the 2009 and the 2017 policies (see Supplementary Table S5). A more focused analysis found that the alcohol policy effects significantly improved the model fit for each age group except for those 40–49 years of age (as shown in Table 2). Thus, the two alcohol policy variables explained additional variance in all-cause mortality rates per 100,000 people above and beyond the secular trends identified in the joinpoint analysis.

**Table 1:** Joinpoint location, confidence interval and trend of declining segment by age group.

Age groups	Joinpoint 1 Cumulative month (95% CI) and corresponding year		Joinpoint 2 Cumulative month (95% CI) and corresponding year		Joinpoint 3 Cumulative month (95% CI) and corresponding year		Declining segment slope *b*	Slope t-value and *p* value
20+	72 (53–88)	2007	N/A	N/A	N/A	N/A	− .002	t(212) = − 9.98, *p* < .0001
20–29	77 (52–95)	2007^a^	N/A	N/A	N/A	N/A	− .005	t(212) = − 10.7, *p* < .0001
30–39	26 (3–97)	2003	81 (70–138)	2007^a^	101 (83–202)	2009	− .013	t(208) = − 2.45, *p* = .015
40–49	83 (11–89)	2007^a^	112 (71–115)	2010	115 (85–214)	2010	− .013	t(208) = − 5.79, *p* < .0001
50–59	85 (78–96)	2008^a^	101 (89–112)	2009	N/A	N/A	− .014	t(210) = − 2.24, *p* = .026
60–69	73 (61–86)	2007^a^	N/A	N/A	N/A	N/A	− .002	t(212) = − 10.81, *p* < .0001
70–79	N/A	N/A	N/A	N/A	N/A	N/A	− .001	t(214) = − 7.76, *p* < .0001
80+	N/A	N/A	N/A	N/A	N/A	N/A	− .001	t(214) = − 3.92, *p* < .0001

Joinpoint locations are number of months since the beginning of the dataset (e.g., January 2001 = month 1).

^a^Declining slope segment.

**Table 2:** Comparison of joinpoint model with and without alcohol policies with Likelihood ratio test of significance (two-tailed, *p* value), by age group.

Age groups	Joinpoint with policies		Joinpoint without policies		*p* value
	AIC	R^2^-adjusted	AIC	R^2^-adjusted	
20+	1255.932	0.854	1268.47	0.837	*p* = .0003
20–29	868.94	.76	874.78	.75	*p* = .0073
30–39	1029.55	.68	1033.64	.67	*p* = .018
40–49	1203.73	.80	1204.53	.80	*p* = .091
50–59	1412.38	.87	1417.80	.87	*p* = .0090
60–69	1607.69	.80	1613.06	.80	*p* = .0092
70–79	1919.41	.76	1936.84	.74	*p* < .0001
80+	2490.16	.69	2531.68	.68	*p* = .0001

Lower AIC indicates a better model fit.

In support of Hypothesis 2, when separated by age groups, the models showed a reduction in all-cause mortality rates for those 20–29 years of age following the 2009 alcohol control policy changes. The policy model predicted fewer relative deaths among those 20–29 years of age in the 12-month period following policy implementation (-7.70%, or 1 death per 100,000 people; see Table 3) compared to the joinpoint model alone. In further support of Hypothesis 2, for those 30–39 years of age, the models suggest a significant reduction of all-cause mortality rates following the 2017 alcohol control policy changes, with an estimated 6.5% decrease in all-cause mortality rates (or 0.5 deaths per 100,000 people). In addition, we found a significant effect of the 2009 policy for those 70–79 years of age (see Table 3), however because the purpose of this article is to investigate premature death, we will not discuss this finding further.

**Table 3:** Age-specific effects of the alcohol control policies implemented in 2009 and 2017, and the effect on monthly all-cause mortality rate (per 100,000 people) for the 12-month period following policy implementation.

Age groups	Policy 2009				Policy 2017			
	Model 1: joinpoint analysis	Model 2: model 1 + policy effect	Relative change model 1 versus model 2, % difference (95% CI)	t-value and *p* value of 2009 policy in model 2	Model 1: joinpoint analysis	Model 2: model 1 + policy effect	Relative change model 1 versus model 2, % difference (95% CI)	t-value and *p* value of 2017 policy in model 2
20–29	12.23	11.35	− 7.70% (− 8.24 to − 6.14)	*t*(212) = − 3.05, *p* = .0026	7.28	7.27	0.16% (0.12–0.16)	*t*(212) = − 1.193, *p* = .23
30–39	23.21	23.12	− 0.37% (− 0.40 to − 0.29)	*t*(212) = − 0.669, *p* = .50	17.78	16.69	− 6.49% (− 7.45 to − 5.870)	*t*(212) = − 2.554, *p* = .011
40–49	46.25	46.38	0.28% (0.22–0.30)	*t*(212) = 0.062, *p* = .95	35.26	34.61	− 1.91% (− 3.16 to − 2.28)	*t*(212) = − 0.85, *p* = .40
50–59	96.83	96.48	− 0.36% (− 0.42 to − 0.33)	*t*(212) = − 0.42, *p* = .68	71.59	69.35	− 3.24% (− 3.92 to − 1.90)	*t*(212) = − 1.39, *p* = .16
60–69	186.45	185.18	− 0.68% (− 0.84 to − 0.65)	*t*(212) = − 0.76, *p* = .45	150.61	148.42	− 1.48% (− 1.52 to − 1.12)	*t*(212) = − 1.26, *p* = .21
70–79	343.52	332.65	− 3.26% (− 3.67 to − 2.77)	*t*(212) = − 3.67, *p* = .00030	307.43	309.15	0.56% (0.48–0.64)	*t*(212) = − 0.83, *p* = .40
80 +	1025.96	1022.85	− 0.30% (− 0.33 to − 0.25)	*t*(212) = − 0.27, *p* = .78	933.96	914.09	− 2.17% (− 2.26 to − 2.66)	*t*(212) = − 0.78, *p* = .43

In addition, an exploratory sex-specific analysis showed that these policy effects were driven by males rather than females (see Supplementary Figures SF18–SF24, Table S4). We also converted the t-score of the policy effect in 2009 for those 20–29 years old and the policy effect in 2017 for those 30–39 years old into a standardized effect size (*r* = − 0.20 and r = ﻿− 0.17 respectively). We computed the absolute number of deaths for those 20–29 years old in 2009 (610 deaths) and with such an effect size (R^2^-adjusted = 0.04) the alcohol control policy measures would have amounted to avoiding 24 deaths. The yearly absolute number of deaths in those 30–39 years old in 2017 was 688, and such an effect size (R^2^-adjusted = 0.029) for alcohol control policy would have amounted to avoiding 20 deaths.

## Discussion

Our findings support both our hypotheses. With respect to Hypothesis 1, in the aggregated adult group (20+ years of age), the alcohol control policies resulted in a significant reduction in all-cause mortality when controlling for secular trends using the joinpoint analysis. Furthermore, as per Hypothesis 2, for those 20–29 years old the 2009 alcohol control policy measures were most impactful, and for those 30–39 years old the 2017 alcohol policy was most impactful. The policies implemented in 2009 and 2017 added significant explanatory power to the joinpoint model across virtually all of the age groups (average increase of 1% variance), lending support for a causal interpretation of the findings by Štelemėkas et al.^32^. This study strengthens the argument that alcohol control policies should be an important consideration when aiming to reduce all-cause mortality.

We employed a two-stage data analysis strategy to control for and model counterfactual events. In natural experiments and quasi-experimental studies, a primary challenge is identifying an appropriate control condition^35^. Using a data-driven approach, the joinpoint analysis identified macro-level linear trends, which were used as a base model in the present study to directly compare the effect of key independent variables (alcohol control policies introduced in 2009 and 2017). Future research may employ a similar two-stage technique to evaluate the effectiveness of this strategy as a means of creating an adequate base model or control condition compared to other possible methods.

The current study presents further evidence that alcohol control policies impact age groups differently^11,22,24,25^. The largest effects were observed among young adults (20–29 and 30–39 years of age) exhibiting a high relative effect of alcohol policy in reducing mortality rate (about 7% reduction in their mortality rate). The 20–29 and 30–39 age groups have a high prevalence of HED (40–45%), as discussed above. Given that the effects of alcohol policy were found in these age groups, we speculate that HED may have been an underlying behavior affected by the alcohol control policies. In contrast, older adults had little change in mortality rate as a result of the alcohol control policies, when controlling for secular trends, supporting the finding that alcohol may pose varying levels of risk to all-cause mortality depending on the age group (we did find a significant effect of the 2009 policy in the 70–79 age group, however as discussed above, there are many factors that contribute to all-cause mortality in this group and because alcohol is generally a low risk factor, it was likely that it was a spurious finding)^18^. Young adults have a relatively low absolute mortality rate (7.27–23.21 deaths per 100,000 people; see Table 3) whereas generally older adults have much higher mortality rates (34.61–1025.96 deaths per 100,000 people; see Table 3). Therefore preventing even a small number of deaths in young adults has important implications for global health and the UN Sustainable Development Goals initiative for reducing premature death^36^.

Our findings are in apparent contradiction of previous work investigating alcohol pricing policies and mortality^22^. We note that our approach, unlike past work, was aimed at controlling for underlying trends specific to different age groups. These linear trends (jointpoint analysis) explained a significant amount of mortality rate variance and allowed for a unique focus on the immediate effects of alcohol control policies. Gradual, longitudinal effects are more challenging to model using this methodology as chronic causes of mortality vary in long-term trends and may be obscured by the linear segments. For instance, alcoholic liver cirrhosis measured at a population level follows a combination of immediate and delayed effects where changes in consumption affect mortality within the first 2 years^37,38^. In our study, we focused on all-cause mortality and modelled our effects as abrupt and permanent for the alcohol policies of interest. Although some chronic conditions may still be amenable to this methodology (e.g., the immediate effects on liver cirrhosis), future work should look at long-term impact of alcohol control policies and its effect on chronic disease. Compared to past work, our method of controlling for secular trends along with the interrupted time series analysis methodology provides a strong inference of causality. In addition, in contrast to past work of alcohol policy and age-groups, we intentionally investigated all-cause mortality—an indicator of overall public health. The findings of this study highlight how alcohol control policy may have a greater impact on young adults and draws attention to the role of alcohol consumption in all-cause mortality in age groups of 20–29 and 30–39 years.

The policies studied in this paper are significant because they fall under the umbrella of the World Health Organization’s “best buys” (see Štelemėkas et al.^32^ for full policy details), which are most effective and cost-effective to implement, with respect to changing health outcomes. In addition, Lithuania is a member of the European Union, located in the Baltic region of Europe; it is a high-income country, with strong civil liberties, and a population of 2,794,184 in 2019. These characteristics make the country an ideal candidate for drawing conclusions that extend to comparable, well-developed high-income western societies. Furthermore, we demonstrate that alcohol policies can impact various subgroups of the population differently. Understanding these specifics will improve policy development by aiding in matching country characteristics to optimal outcomes. In some countries, there exists vulnerable groups of individuals and wide economic disparities, which result in some being more prone to alcohol-related harm. Identifying the most effective means of preventing all-cause mortality and premature death for these vulnerable groups may be of interest for policy makers and for the overall development of any country^26^.

This study has some limitations worth noting, however. Firstly, the legal drinking age in Lithuania was 18 years up until January 2018, however due to the nature of our data (10-year age groups) we could not analyze data for individuals 18 years and older. We did, however, conduct a sensitivity analysis on individuals 10+ years of age and found similar results as in our analyses with individuals 20+ years of age, such that the policy model explained significantly more variance, and that the policies implemented in 2009 and in 2017 were both significant (see Supplementary Materials). Second, the policy effects cannot be completely isolated from the secular trends, and in fact due to the close temporal overlap, it could be that the joinpoint analysis removed variance related to the policy effects. Given that the joinpoint analysis is data-driven, it cannot distinguish between potential overlapping effects of the secular trends (e.g., economic and political) and therefore the alcohol control policy effects may have been underestimated. Other strategies, such as fitting a cubic trend, or a smoothing function with fewer knots may be an alternative method of controlling for underlying trends and could be tested against a joinpoint analysis in future studies. Also, the modelling approach of this study is not well-suited for observing the long-term impact, or the interaction effect of these policies. Some have found that policies in place during adolescence can have distal effects on consumption patterns in adulthood^39^. Consequently, due to the nature of our approach, testing the effect of policies on mortality in older adults may have been underestimated as they are more likely to die due to chronic conditions. We also note that our study focuses on the relative change in mortality rates within each age group rather than absolute mortality. That is, there was a much higher number of absolute deaths in older adults (e.g., 93.68 deaths per 100,000 adults aged 50–59 in 2009, whereas there were 11.56 deaths per 100,000 adults aged 20–29 in 2009), which would have decreased to a greater degree as a raw rate, following the policy implementation. However, the focus of the current study was on the effect size of the *relative* change in deaths following policy implementation. Furthermore, as discussed below, the joinpoint analysis was set to a maximum of three joinpoints. This parameter resulted in a model (for most cases) which mapped the decline from 2007 to 2008 onward as a single linear trend. However, there were several alcohol policies implemented during this time and it is likely that other secular events also occurred during this period. The cumulative effect and perhaps the smaller fluctuations (increases or decreases) in mortality, may not have been adequately captured with a simpler joinpoint model that contained only 3 joinpoints for the secular trends. One might also expect that our effects were strongest for alcohol-attributable mortality rates, however the alcohol-related death data from Lithuania over the study period were relatively unreliable due to the coding practices only becoming up-to-date recently and thus, it was a limitation that we were unable to do an additional analysis focused on alcohol-attributable causes of death^40^. Lastly, other cultures (such as those in Eastern Mediterranean Region, South East Asian Region) demonstrate markedly different alcohol consumption patterns^12,13^ and therefore, the effect of alcohol control policy on alcohol consumption and all-cause mortality may differ in these areas.

Overall, however, this study lends indirect support to the existing literature linking alcohol consumption to all-cause mortality; that is, when stricter alcohol control policies were introduced (and presumably alcohol consumption decreased), there was a decrease in all-cause mortality rates^9^. The current study adds to the literature by showing that even when controlling for larger secular trends alcohol control policy can still have a considerable impact on all-cause mortality rates. Furthermore, this study identifies a larger effect of alcohol control policies on all-cause mortality in certain age groups (young adults, 20–29 and 30–39 years of age) where policies may play a role in preventing premature death. This paper adds support for the use of alcohol control policy in decreasing all-cause mortality and adds to our understanding of who is most affected by alcohol control policies.

## Method

### Dataset

The dataset used in the present study was obtained from Statistics Lithuania (for data from 2001 to 2009) and Lithuanian Institute of Hygiene (for data from 2010 to 2018), which contained the absolute number of all-cause deaths aggregated by 10-year age groups: 0–9, 10–19, 20–29, 30–39, 40–49, 50–59, 60–69, 70–79, and 80+ years. The dataset contains mortality data over the course of 18 years, from January 2001 – December 2018 (*n* = 216 months).

### Mortality Rate

The dependent variable examined was the aggregated age-standardized (20+) and age group specific all-cause mortality rate, defined as number of deaths per 100,000 people in Lithuania. Monthly data on absolute all-cause deaths were combined with population data in Lithuania to compute mortality rates, i.e., monthly deaths per 100,000 people*.* Specifically, we calculated age-standardized (20+ years of age) as well as age group specific (20–29, 30–39, 40–49 years of age etc.) all-cause mortality rates for each of the 216 months combined for both sexes as per the following formula:

$$\frac{d}{n} \times 100{,}000$$

where *d* = the deaths from all-causes and *n* = total population per age group. Given that the purpose of the study was to examine alcohol control policy effects, and the legal drinking age is 20 in Lithuania, only adults 20 years of age and older were included in the study.

In addition, as part of a sensitivity analysis, we also analyzed liver cirrhosis mortality rates (liver cirrhosis deaths per 100,000 people) for those 20+ using the same methodology, as well as implementing a lag structure (see Supplementary Materials for analyses and full description).

### Statistical analyses

To address Hypothesis 1 and demonstrate that alcohol control policies can explain variance in addition to secular trends, the statistical analyses were carried out in two subsequent steps. To address Hypothesis 2 and show that the effects were largest among young adults (20–29 years of age and 30–39 years of age) this technique was repeated for each age group separately. First, joinpoint regression analyses were performed in order to determine secular trends across the time series of all-cause mortality rates between 2001 and 2018 and to serve as a base model. The joinpoint analysis is a data-driven statistical technique which identifies inflection points in the data and fits various linear regression lines based on a pre-selected number of joinpoints^41^. The fact that the data are used to derive the linear regression segments makes it an ideal technique to control for unknown secular trends that occurred during the time period of the present dataset. From these joinpoint models, the predicted monthly all-cause mortality rates were obtained and included as covariate in subsequent models. Second, generalized additive mixed models (GAMM) were employed to estimate the effect size of the two sets of alcohol control policies in 2009 and 2017 while covarying for the joinpoint regressions, which controlled for secular trends in the data.

The joinpoint analyses were conducted using the Joinpoint Regression Program, version 4.8.0.1 (Statistical Research and Applications Branch, National Cancer Institute, http://srab.cancer.gov/joinpoint/). The joinpoint analysis identifies inflection points in the data and confidence intervals for separate segments, while also controlling for autocorrelation. For the present analyses a maximum of three joinpoints was specified, based on a visual inspection of the data and based on the previous analysis that indicated two significant policy effects on all-cause mortality (see Štelemėkas et al.^32^). Based on the maximum number of joinpoints, linear segments were fitted to the data. Using a Monte Carlo Permutation method, the fewest number of linear segments such that an additional joinpoint does not add a statistically significant linear trend is selected^41^. The predicted values from the joinpoint analyses were then used as a covariate in the subsequent GAMM, to control for secular trends (see Figs. 1 and 2, and Supplementary Figures SF1-SF6 and Figures SF15–SF21, for the use of GAMM see Beard et al.^42^). Including these values in the final model removes variance explained by the underlying secular trends in mortality rates when using the GAMM and is thus superior to simply adding a continuously ascending time variable (e.g., months). In addition, dummy-coded variables representing policy effects for 2009 and for 2017 (coded 0 for years preceding the policy, and 1 for the months following policy implementation) were included in the GAMM. In the GAMM seasonality was adjusted for using smoothing splines with 12 knots (monthly pattern). To determine whether the inclusion of the alcohol policies improved the model, the models were compared with and without policy effects. An omnibus likelihood ratio test was used to compare our two competing models, Model 1: which included the joinpoint values as a predictor variable, while controlling for autocorrelation and seasonality, and Model 2: which included the joinpoint values as a predictor variable as well as the two dummy coded policy variables, while also controlling for autocorrelation and seasonality. All analyses were performed using R version 4.0.2.

## Supplementary Information

Supplementary Information.

## Data Availability

The original data are administrative data of the Lithuanian government agencies, and need to be obtained directly from the original source (exact sources as indicated in the article). The R code used to analyze and compute variables can be found in the supplementary materials on the web.
