# Supplementary material for: Alcohol control policies add to secular trends in all-cause mortality rates in young adults
Source: Sci Rep. 2021 Jul 23;11:15127. doi: 10.1038/s41598-021-94562-1 (PMC8302690; doi:10.1038/s41598-021-94562-1)
Supplement: Supplementary file 1 — Supplementary Information. [file 41598_2021_94562_MOESM1_ESM.docx]

# Alcohol control policies add to secular trends in all-cause mortality rates in young adults

Alexander Tran^1*^, Jakob Manthey^2-4^, Shannon Lange^1^, Huan Jiang^1,7^, Mindaugas Štelemėkas^10,11^, Vaida Liutkutė – Gumarov^10^, Olga Meščeriakova-Veliulienė^12^, Janina Petkevičienė^10,11^, Ričardas Radišauskas^13,14^, Tadas Telksnys^10^, Jürgen Rehm^1,2, 5-9^

^1^ Institute for Mental Health Policy Research, Centre for Addiction and Mental Health (CAMH), 33 Russell Street, Toronto, Ontario, Canada, M5S 2S1

^2^ Institute of Clinical Psychology and Psychotherapy, Technische Universität Dresden, Chemnitzer Str. 46, 01187 Dresden, Germany

^3^ Center for Interdisciplinary Addiction Research (ZIS), Department of Psychiatry and Psychotherapy, University Medical Center Hamburg-Eppendorf (UKE), Martinistraße 52, 20246 Hamburg, Germany

^4^ Department of Psychiatry, Medical Faculty, University of Leipzig, Semmelweisstraße 10, 04103 Leipzig, Germany

^5^ Campbell Family Mental Health Research Institute, CAMH, 250 College Street, Toronto, Ontario, Canada, M5T 1R8

^6^  Department of Psychiatry, University of Toronto, 250 College Street, 8th Floor, Toronto, Ontario, Canada, M5T 1R8

^7^  Dalla Lana School of Public Health, University of Toronto, 155 College Street, 6th Floor, Toronto, Ontario, Canada, M5T 3M7

^8^ Institute of Medical Science (IMS), University of Toronto, Medical Sciences Building, 1 King’s College Circle, Room 2374, Toronto, Ontario, Canada, M5S 1A8

^9^ Department of International Health Projects, Institute for Leadership and Health Management, I.M. Sechenov First Moscow State Medical University, Trubetskaya str., 8, b. 2, 119992, Moscow, Russian Federation

^10^ Health Research Institute, Faculty of Public Health, Lithuanian University of Health Sciences, Tilžės 18, 44307 Kaunas, Lithuania

^11^ Department of Preventive Medicine, Faculty of Public Health, Lithuanian University of Health Sciences, Tilžės 18, 44307 Kaunas, Lithuania

^12^ Department of Health Management, Faculty of Public Health, Lithuanian University of Health Sciences, Tilžės 18, 44307 Kaunas, Lithuania

^13^ Department of Environmental and Occupational Medicine, Faculty of Public Health, Lithuanian University of Health Sciences, Tilžės 18, 44307 Kaunas, Lithuania

^14^ Institute of Cardiology, Lithuanian University of Health Sciences, Sukilėlių av. 15, 50162, Kaunas, Lithuania

*Corresponding author

Alexander Tran

alexander.tran@camh.ca

# SUPPLEMENTARY MATERIAL

**Sensitivity Analysis: Liver Cirrhosis Mortality rate (20+, age-standardized)**

In order to test the effects on alcohol-specific cause of death, we conducted the same analysis on liver cirrhosis deaths. In addition, according to previous research, [35,36] the effects of alcohol consumption on liver cirrhosis occur in a lagged manner. Thus, we compared our dummy coding of the policy effects to a lagged structure, in which we created variables that achieved 60% effect within the first month, 80% effect in the next 11 months, 90% in the next 12 months, and 100% effect onward.

The model statistics of the analyses can be found below, which indicate that the Policy 2017 was marginally significant for liver cirrhosis deaths (*p* = .054), however Policy 2009 was not significant (*p* = .78). Furthermore, the effect of the policies were generally similar between the lag structure policy variables (Policy 2017, *p* = .058; Policy 2009, *p* = .68) and the dummy coded version. Both the lag structure model and the dummy coded model had the same fit (R^2^-adjusted = .824). Thus, our analyses indicate that the alcohol policies affected alcohol-attributable mortality (LC) although it appeared to be stronger for all-cause mortality.

## **Supplementary Table S1.** Dummy coded and Lag structure policy effects on age-standardized liver cirrhosis mortality rates (aged 20+)

| Model parameters | Intercept (95% CI) | Joinpoint covariate(95% CI) | Policy 2009 (95% CI) | Policy 2017 (95% CI) |
| --- | --- | --- | --- | --- |
| Dummy coded policies | **0.11**  **(8.98 to 55.22)** | **0.97*****  **(0.33 to 0.90)** | **0.01**  **(-8.89 to -2.06)** | **-0.17†**  **(-6.97 to -0.14)** |
| Lag structure policies | **0.10**  **(8.98 to 55.22)** | **0.97*****  **(0.33 to 0.90)** | **0.02**  **(-8.89 to -2.06)** | **-0.19†**  **(-6.97 to -0.14)** |

Note: **†** *p* < .1, * *p* < .05, ** *p* < .01, *** *p* < .001


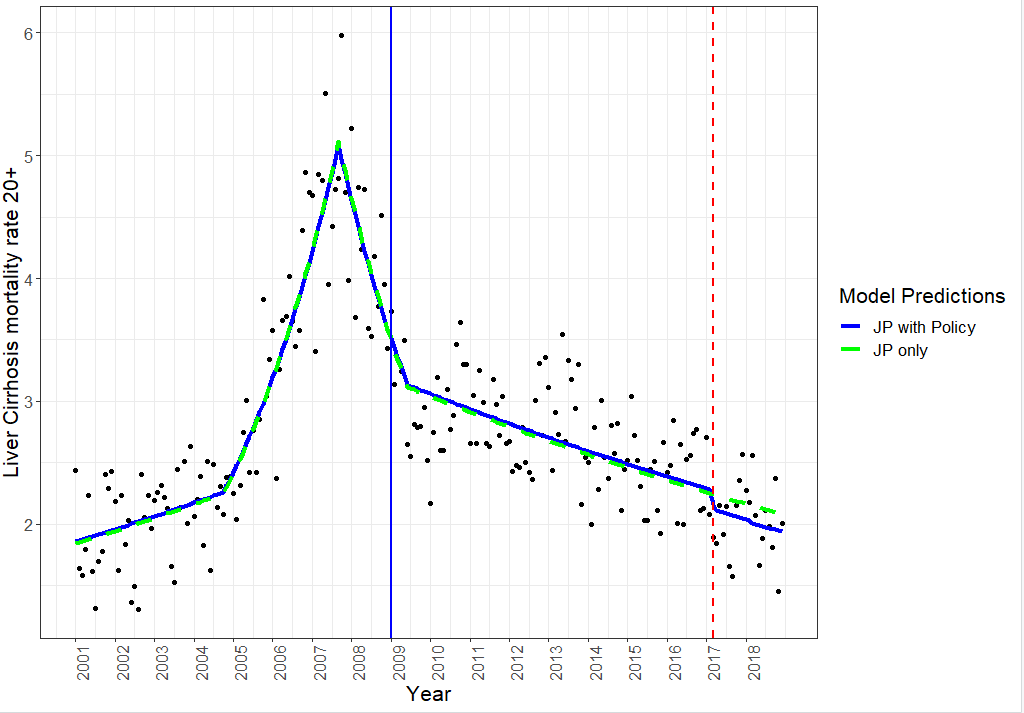


## **Supplementary Figure SF1.** Scatterplot trend of liver cirrhosis mortality rate (deaths per 100,000 people) for ages 20+.

**Sensitivity Analysis: Modelling Policies in 2008, 2009, and 2017 (20+, age-standardized)**

In 2008, Lithuania declared the “Year of Sobriety” in which a number of alcohol control policies were implemented. This was a widely politicized event, and thus the social effects would be difficult to distinguish from the marked changes in alcohol policies. For this reason, it may be difficult to determine what contributed most to reducing all-cause mortality, general secular trends, or the policies in 2008. To attempt to answer this question, as an alternative to the joinpoint analysis to control for mortality trends, we used an interrupted time series analysis that modelled only 3 sets of policies implemented in 2008, 2009, and 2017. The model fit (R2-adjusted = .811) was comparable to the model with the joinpoint analysis and two policies (R2-adjusted = .837), however it was slightly worse. These findings suggest that the secular trends may be better controlled for using the joinpoint analysis.

## **Supplementary Table S2.** Model statistics for ITSA of Policies in 2008, 2009, and 2017

|  | Estimate | Standard Error | t-value | p-value |
| --- | --- | --- | --- | --- |
| Intercept | 101.78 | 1.25 | 81.37 | *p* < .0001 |
| Policy 2008 | -0.85 | 3.52 | -0.24 | *p* = .81 |
| Policy 2009 | -13.96 | 3.47 | -4.02 | *p* < .0001 |
| Policy 2017 | -9.53 | 2.25 | -4.24 | *p* < .0001 |


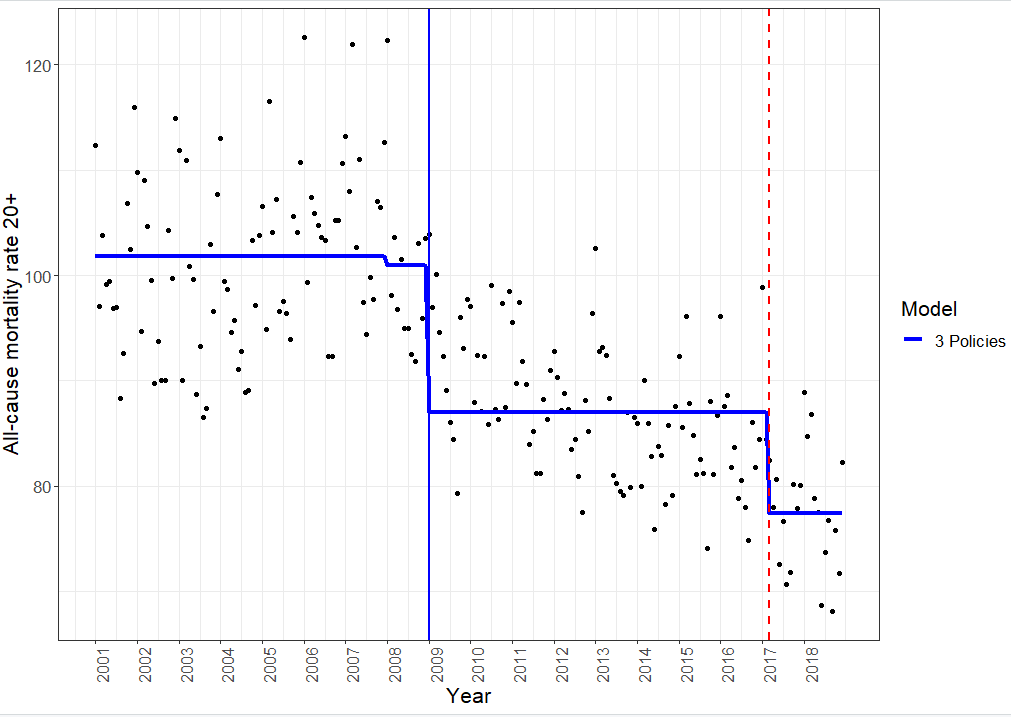


## **Supplementary Figure SF2.** Scatterplot trend of mortality rate (deaths per 100,000 people) for ages 20+ and model of policies in 2008, 2009, and 2017 in blue.

*Note.* Alcohol control policy implemented in 2009 (increased taxation and reduced availability) and 2017 (increased taxation) shown by the blue line and red line, respectively.

## **Supplementary Table S3.** Expected impactful alcohol control policies implemented in Lithuania

| **Date of policy implementation** | **Month #** | **Policy Description** |
| --- | --- | --- |
| January 1, 2009 | 97 | **Increased alcohol taxation:** Excise taxes raised by 10-15%, removal of tax exemptions for alcohol breweries **Reduced alcohol availability**: Restrictions imposed for off-premise sales, ban on open alcohol in vehicles |
| March 1, 2017 | 195 | **Increased alcohol taxation:** Excise taxes raised by 111-112% for wines and beer and 23% for hard liquors |

## **Supplementary Table S4.** Comparison of joinpoint model with and without alcohol policies with Likelihood ratio test of significance (two-tailed, p-value), by age and gender group

| **Groups** | **Joinpoint with policies** | | **Joinpoint** | | **p-value** |
| --- | --- | --- | --- | --- | --- |
|  | **AIC** | **R^2^-adjusted** | **AIC** | **R^2^-adjusted** |  |
| 10+ |  |  |  |  |  |
| Male | 1358.29 | .777 | 1378.56 | .684 | *p* < .0001 |
| Female | 1068.75 | .811 | 1082.05 | .779 | *p* = .0002 |
| Total | 1160.2 | .855 | 1171.87 | .838 | *p* = .0004 |
| 20+ |  |  |  |  |  |
| Male | 1423.497 | 0.865 | 1432.751 | 0.86 | *p* = 0.0013 |
| Female | 1166.098 | 0.811 | 1179.968 | 0.782 | *p* = 0.0001 |
| Total | 1255.932 | 0.854 | 1268.474 | 0.837 | *p* = .0003 |
| 20-29 |  |  |  |  |  |
| Male | 1114.97 | 0.75 | 1124.52 | 0.74 | *p* = 0.0011 |
| Female | 761.65 | 0.25 | 760.31 | 0.25 | *p* = 0.26 |
| Total | 868.94 | .76 | 874.78 | .75 | *p* = .0073 |
| 30-39 |  |  |  |  |  |
| Male | 1266.20 | 0.67 | 1268.57 | 0.67 | *p* = 0.041 |
| Female | 968.76 | 0.21 | 967.30 | 0.21 | *p* = 0.28 |
| Total | 1029.55 | .68 | 1033.64 | .67 | *p* = .018 |
| 40-49 |  |  |  |  |  |
| Male | 1454.31 | 0.74 | 1462.04 | 0.74 | *p* = 0.0028 |
| Female | 1119.38 | 0.44 | 1120.03 | 0.44 | *p* = 0.097 |
| Total | 1203.73 | .80 | 1204.53 | .80 | *p* = .091 |
| 50-59 |  |  |  |  |  |
| Male | 1650.48 | 0.87 | 1662.72 | 0.86 | *p =* .0003 |
| Female | 1339.42 | 0.70 | 1340.59 | 0.71 | *p* = 0.075 |
| Total | 1412.38 | .87 | 1417.80 | .87 | *p* = .0090 |
| 60-69 |  |  |  |  |  |
| Male | 1878.02 | 0.76 | 1885.99 | 0.76 | *p* = 0.0025 |
| Female | 1520.70 | 0.67 | 1526.18 | 0.67 | *p* = 0.0088 |
| Total | 1607.69 | .80 | 1613.06 | .80 | *p* = .0092 |
| 70-79 |  |  |  |  |  |
| Male | 2121.74 | 0.64 | 2140.09 | 0.62 | *p* <.0001 |
| Female | 1887.99 | 0.77 | 1902.66 | 0.75 | *p =* .0001 |
| Total | 1919.41 | .76 | 1936.84 | .74 | *p* < .0001 |
| 80+ |  |  |  |  |  |
| Male | 2634.81 | 0.65 | 2650.05 | 0.65 | *p* = 0.0001 |
| Female | 2468.85 | 0.64 | 2483.84 | 0.66 | *p* = 0.0001 |
| Total | 2490.16 | .69 | 2531.68 | .68 | *p* = .0001 |

Note: Lower AIC indicates a better model fit.

**Supplementary Table S5.** Full Model effects (Joinpoint and Policy effects) for each age group

| Age group | Intercept (95% CI) | Joinpoint covariate(95% CI) | Policy 2009 (95% CI) | Policy 2017 (95% CI) |
| --- | --- | --- | --- | --- |
| 10+ | **32.10****  **(8.98 to 55.22)** | **0.61*****  **(0.33 to 0.90)** | **-5.48****  **(-8.89 to -2.06)** | **-3.56***  **(-6.97 to -0.14)** |
| 20+ | **40.10****  **(11.39 to 68.82)** | **0.61*****  **(0.32 to 0.90)** | **-6.86****  **(-11.05 to -2.66)** | **-4.23***  **(-8.46 to 0.01)** |
| 20-29 | **4.17****  **(1.11 to 7.23)** | **0.72*****  **(0.50 to 0.95)** | **-1.52****  **(-2.51 to -0.54)** | -0.59  (-1.56 to 0.38) |
| 30-39 | 3.05  (-1.54 to 7.63) | **0.89*****  **(0.71 to 1.07)** | -0.38  (-1.49 to 0.73) | **-1.63***  **(-2.89 to -0.37)** |
| 40-49 | 0.36  (-8.23 to 8.94) | **1.00*****  **(0.83 to 1.16)** | 0.06  (-1.98to 2.11) | -0.99  (-3.29 to 1.30) |
| 50-59 | 5.41  (-15.53 to 26.36) | **0.96*****  **(0.76 to 1.15)** | -1.11  (-6.39 to 4.16) | -3.59  (-8.68 to 1.49) |
| 60-69 | **12.81****  **(-19.47to 45.09)** | **0.76*****  **(0.93to 1.11)** | -1.64  (-5.89to 2.61) | -4.01  (-10.27to 2.25) |
| 70-79 | **175.80****  **(64.94 to 286.66)** | **0.53*****  **(0.22 to 0.83)** | **-23.89*****  **(-36.71 to -11.07)** | -5.17  (-17.39 to 7.05) |
| 80+ | 161.17  (-702.21 to 1024.56) | **0.73*****  **(0.04 to 1.41)** | -12.58  (-103.47 to 78.30) | -30.04  (-106.23 to 46.14) |

Note: * *p* < .05, ** *p* < .01, *** *p* < .001


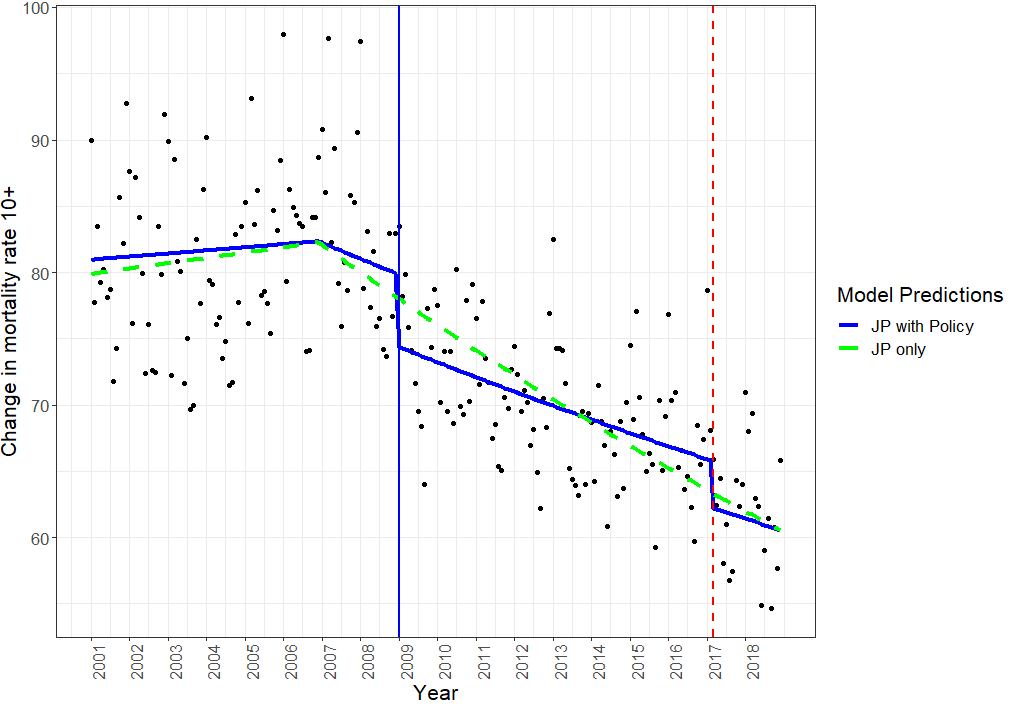


## **Supplementary Figure SF3.** Scatterplot trend of age-standardized mortality rate (deaths per 100,000 people) for ages 10+, both sexes.

*Note.* Alcohol control policy implemented in 2009 (increased taxation and reduced availability) and 2017 (increased taxation) shown by the blue line and red line, respectively.

## **
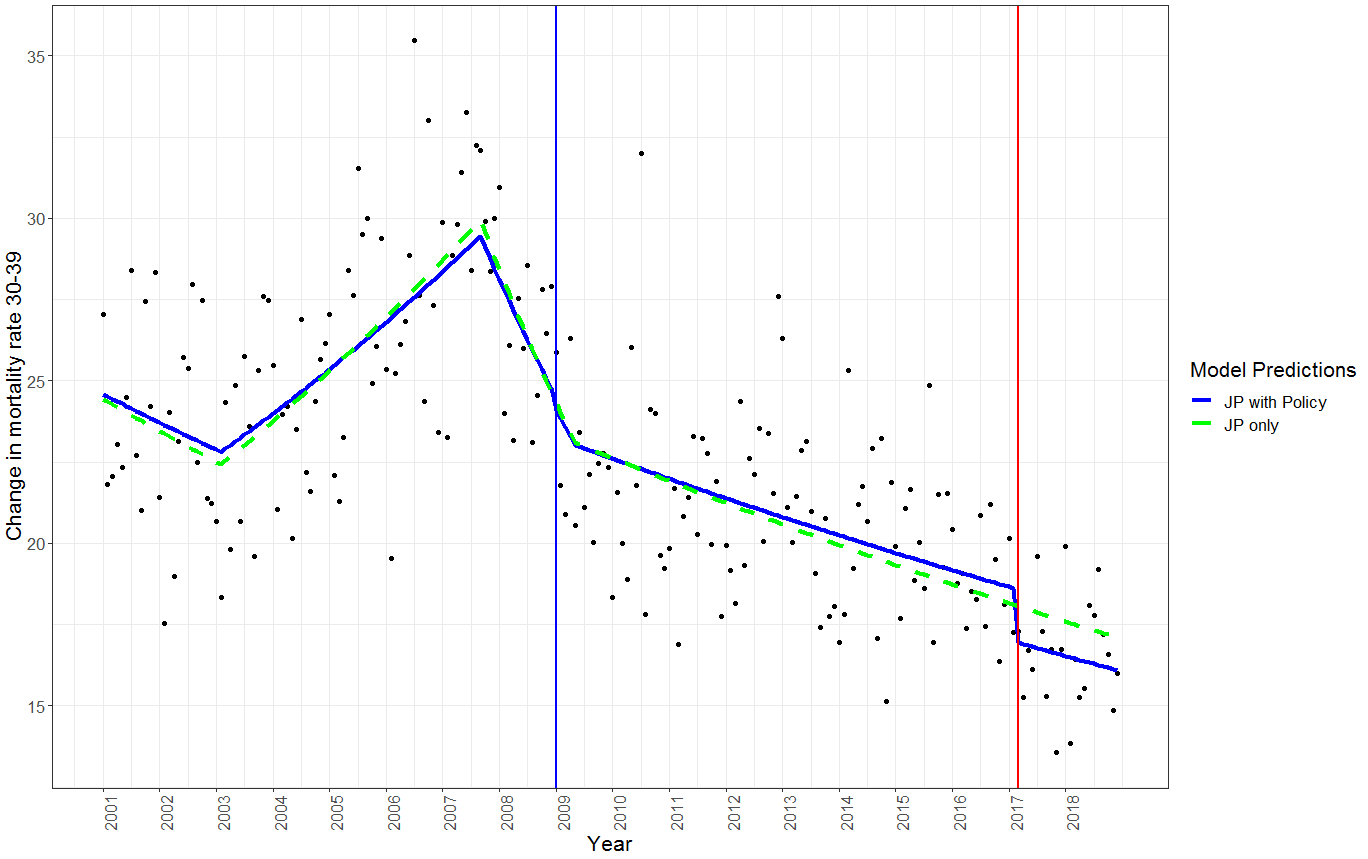
Supplementary Figure SF4.** Scatterplot trend of mortality rate (deaths per 100,000 people) for ages 30-39.

*Note.* Alcohol control policy implemented in 2009 (increased taxation and reduced availability) and 2017 (increased taxation) shown by the blue line and red line, respectively.

## **
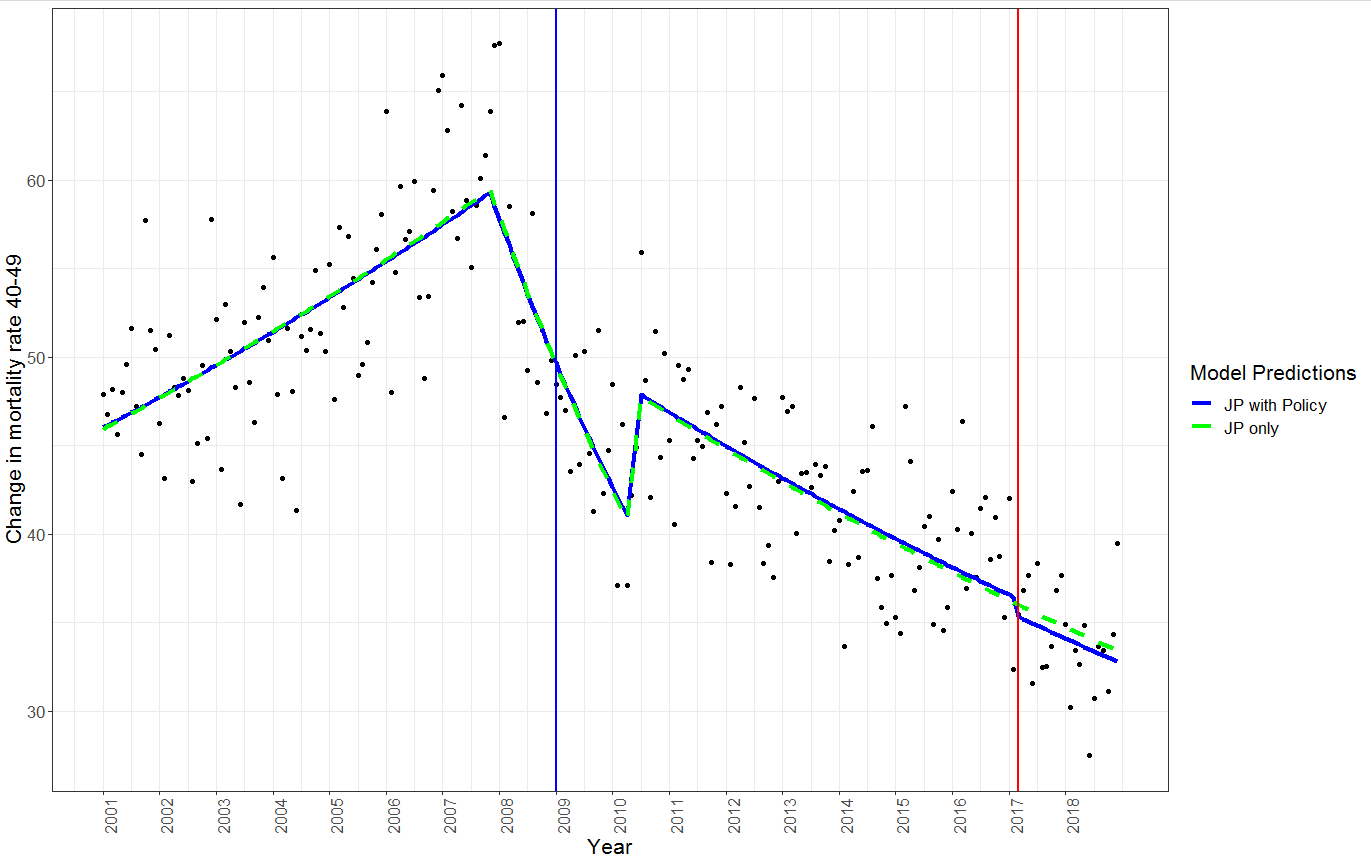
Supplementary Figure SF5.** Scatterplot trend of mortality rate (deaths per 100,000 people) for ages 40-49.

*Note.* Alcohol control policy implemented in 2009 (increased taxation and reduced availability) and 2017 (increased taxation) shown by the blue line and red line, respectively.

##
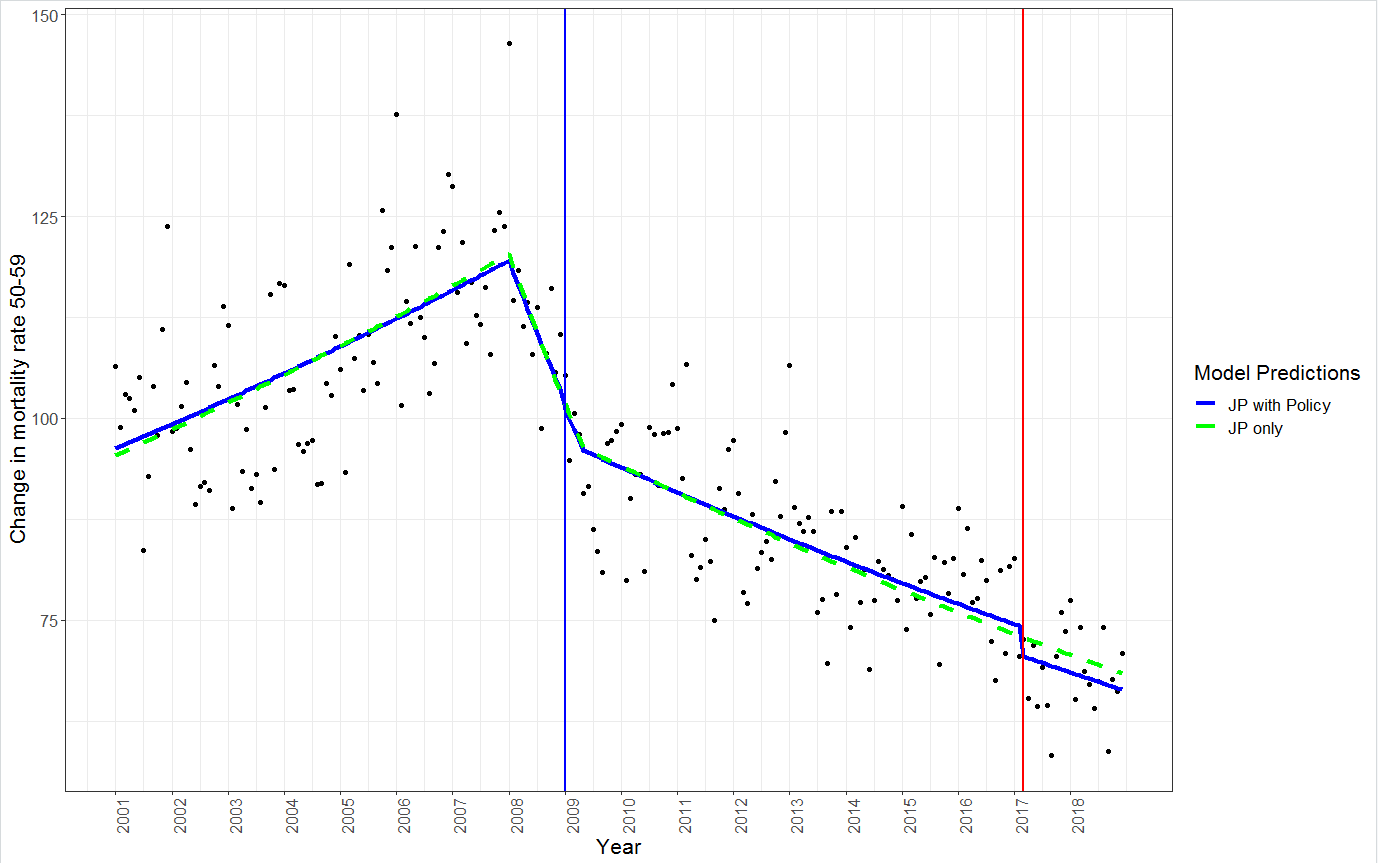
**Supplementary Figure SF6.** Scatterplot trend of mortality rate (deaths per 100,000 people) for ages 50-59.

*Note.* Alcohol control policy implemented in 2009 (increased taxation and reduced availability) and 2017 (increased taxation) shown by the blue line and red line, respectively.

##
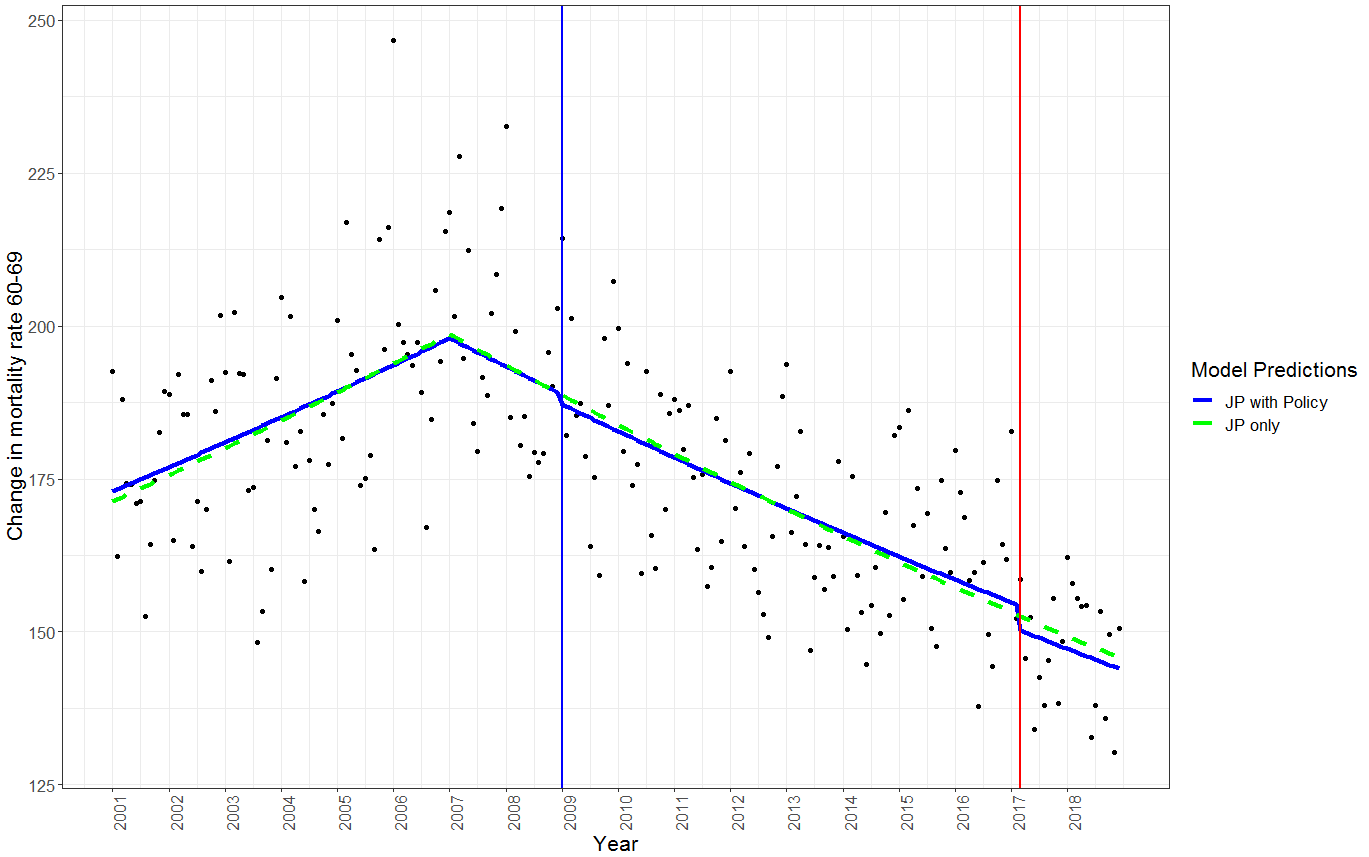
**Supplementary Figure SF7.** Scatterplot trend of mortality rate (deaths per 100,000 people) for ages 60-69.

*Note.* Alcohol control policy implemented in 2009 (increased taxation and reduced availability) and 2017 (increased taxation) shown by the blue line and red line, respectively.

##
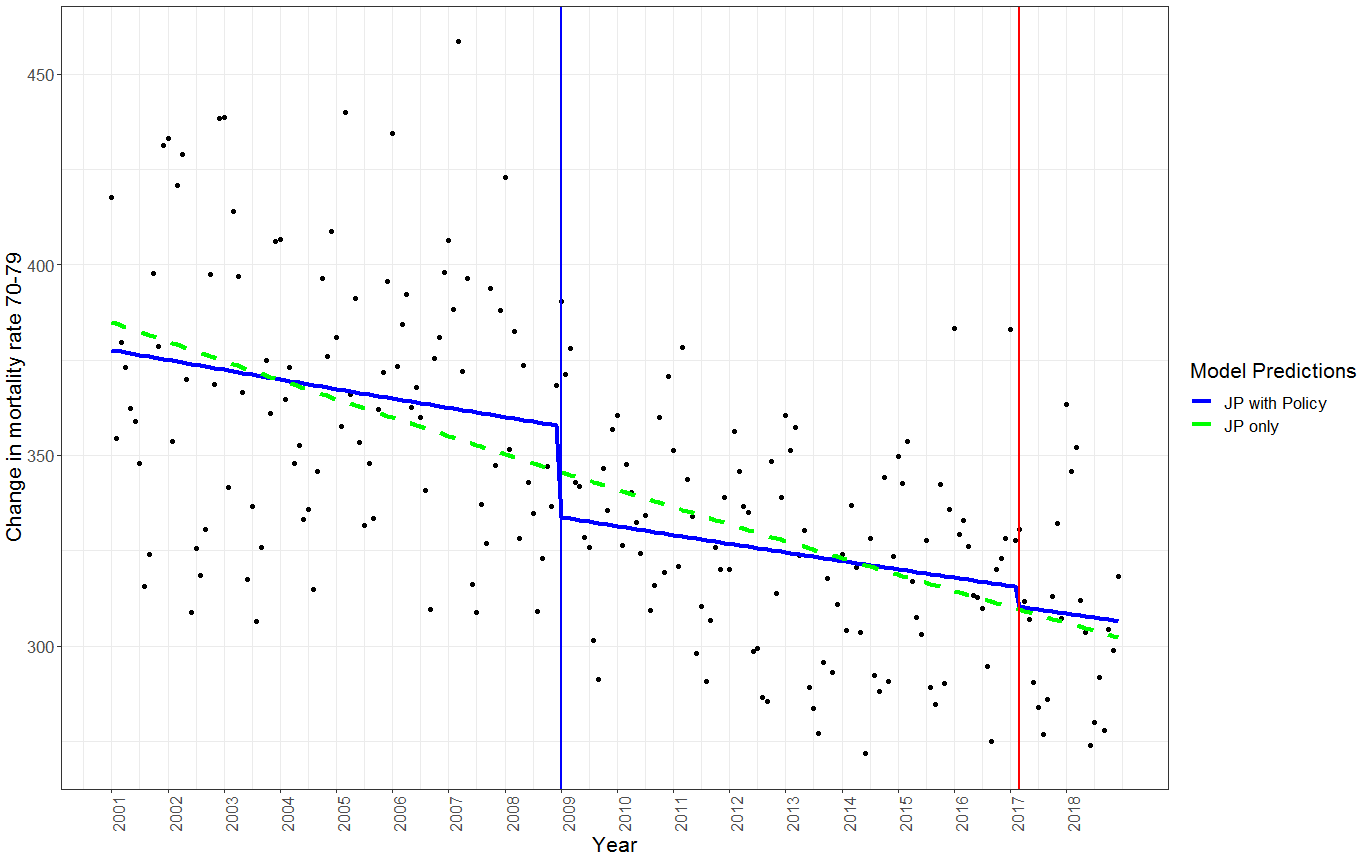
**Supplementary Figure SF8.** Scatterplot trend of mortality rate (deaths per 100,000 people) for ages 70-79.

*Note.* Alcohol control policy implemented in 2009 (increased taxation and reduced availability) and 2017 (increased taxation) shown by the blue line and red line, respectively.

##
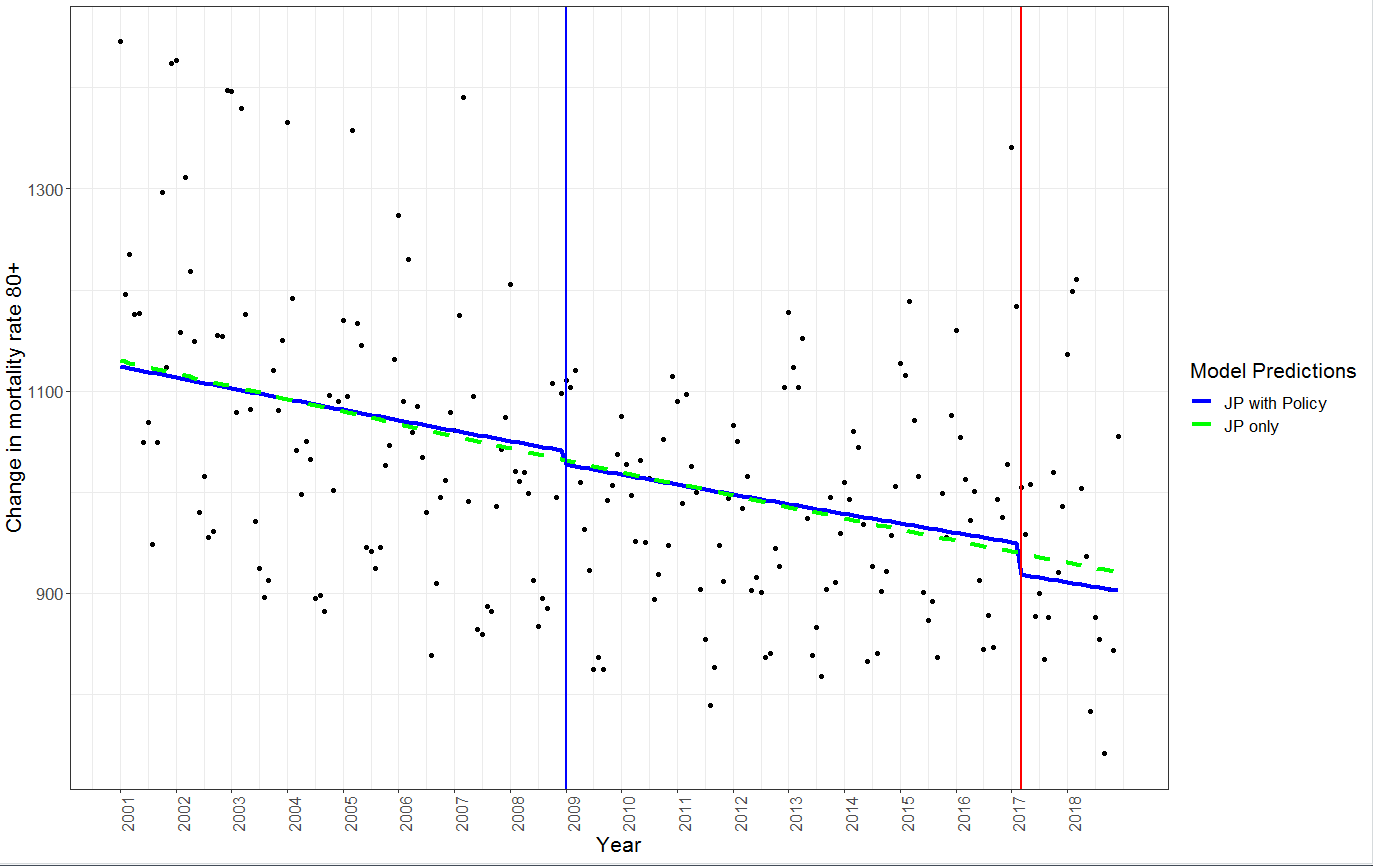
**Supplementary Figure SF9.** Scatterplot trend of mortality rate (deaths per 100,000 people) for ages 80+.

*Note.* Alcohol control policy implemented in 2009 (increased taxation and reduced availability) and 2017 (increased taxation) shown by the blue line and red line, respectively.

##
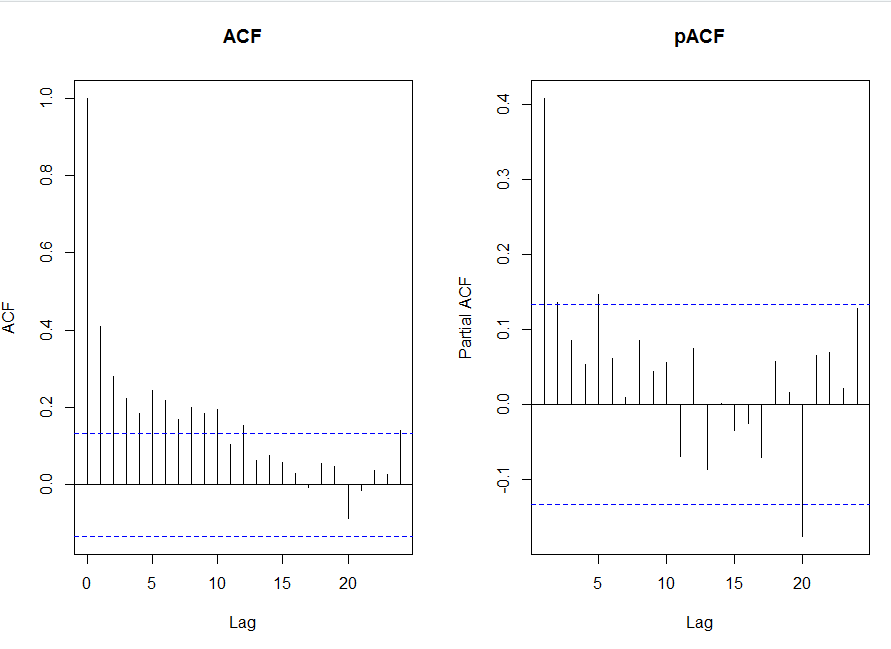
**Supplementary Figure SF10.** ACF and pACF graphs to identify autocorrelation in mortality rate for ages 20+.

##
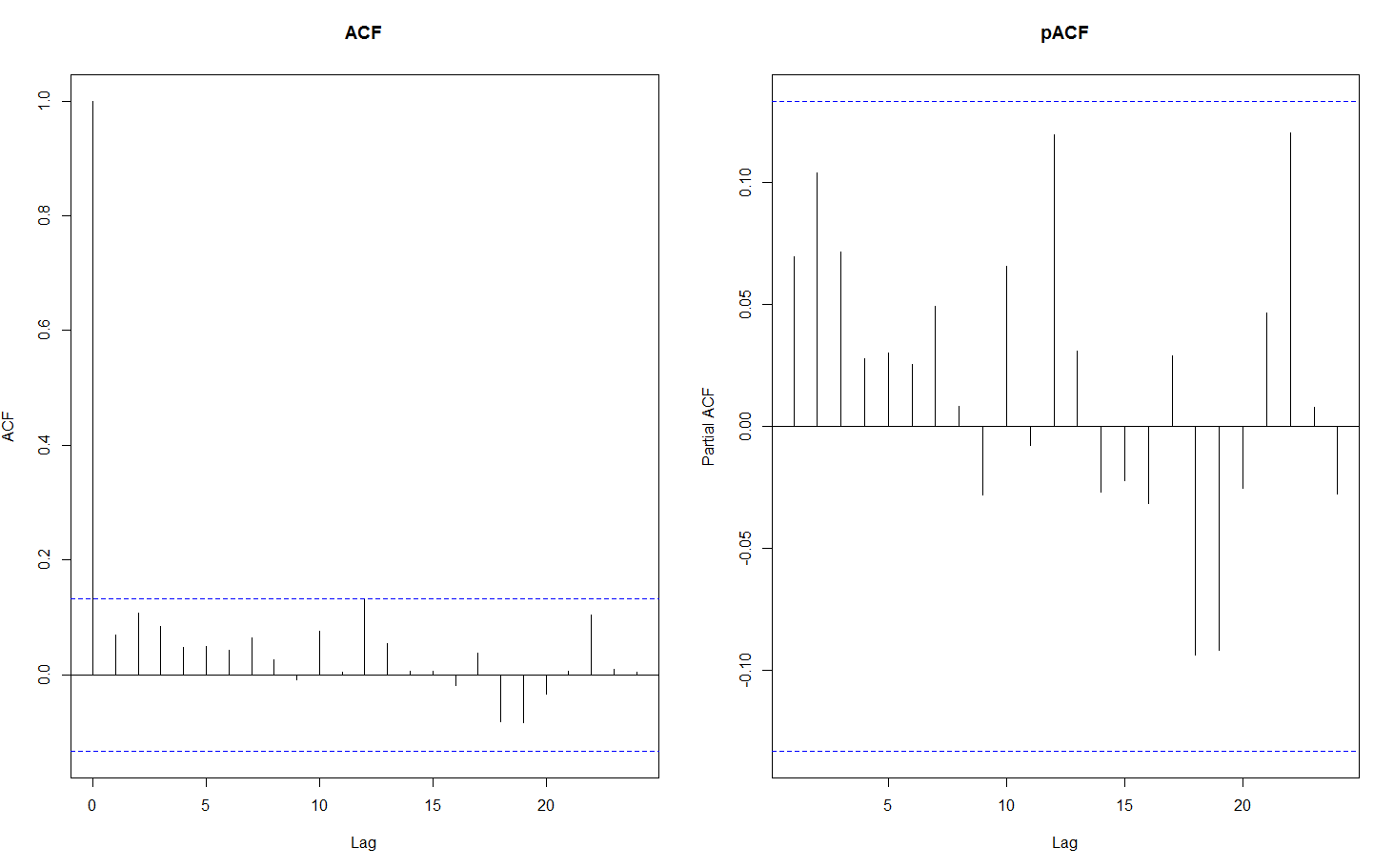
**Supplementary Figure SF11.** ACF and pACF graphs to identify autocorrelation in mortality rate for ages 20-29.

##
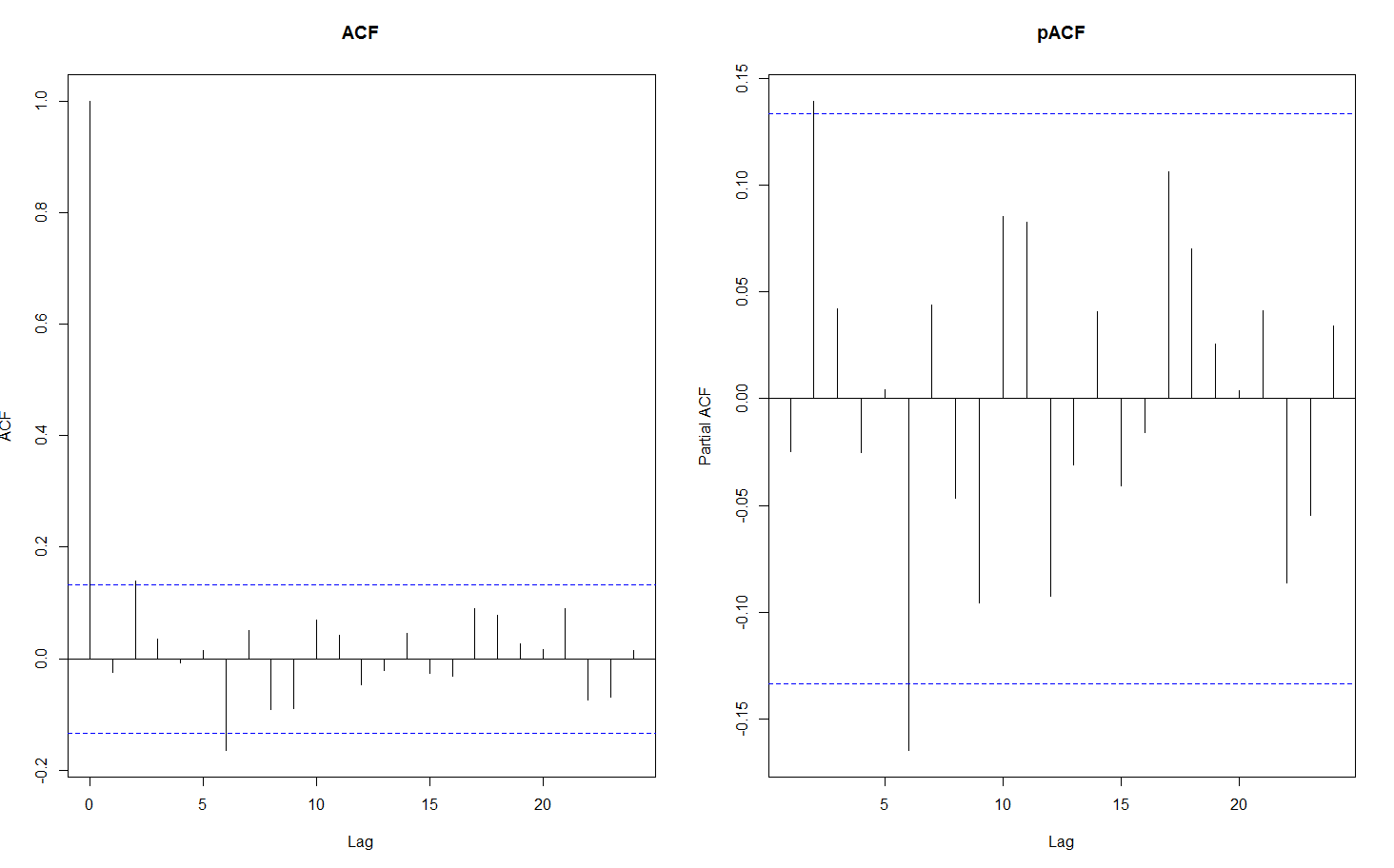
**Supplementary Figure SF12.** ACF and pACF graphs to identify autocorrelation in mortality rate for ages 30-39.

##
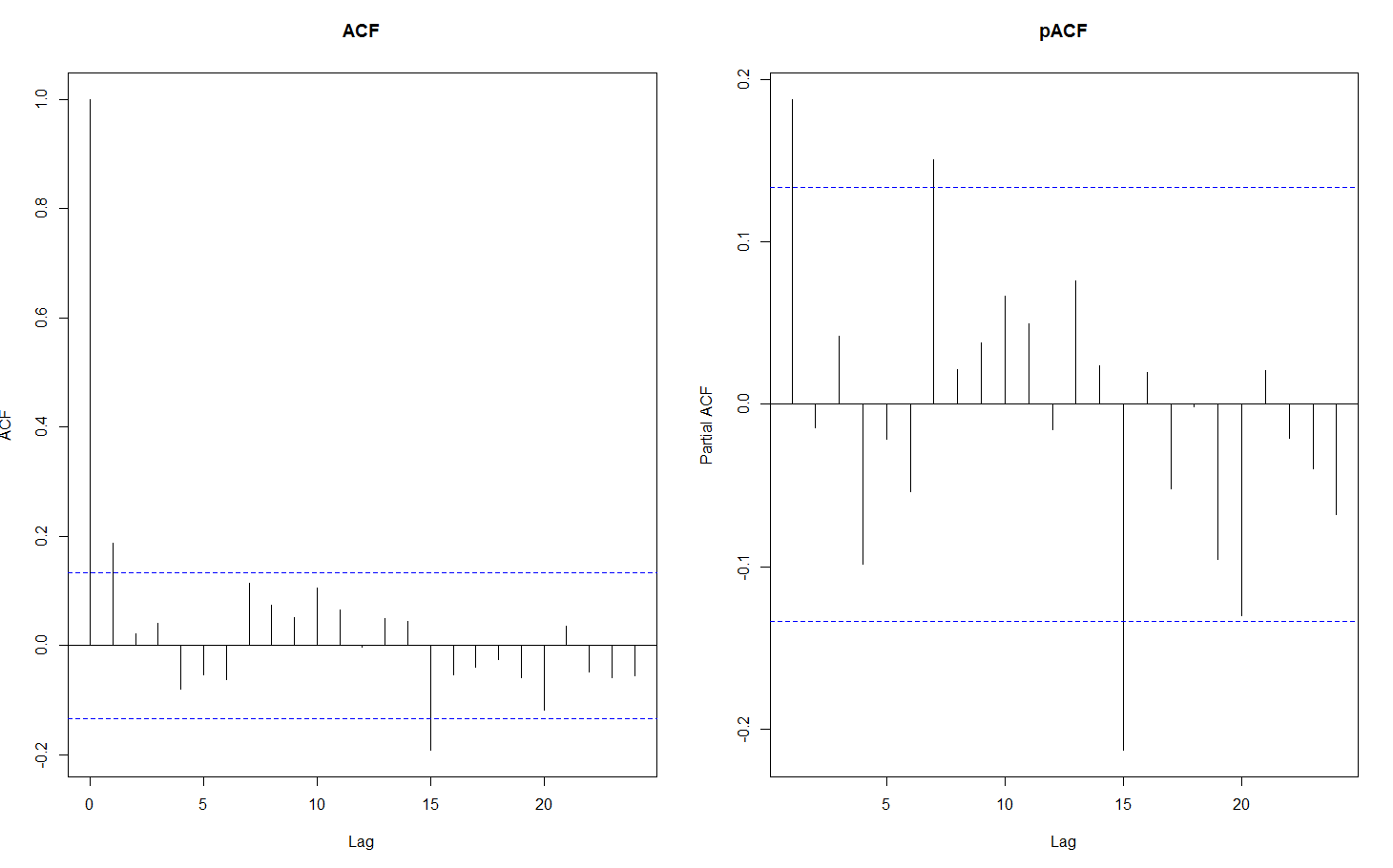
**Supplementary Figure SF13.** ACF and pACF graphs to identify autocorrelation in mortality rate for ages 40-49.


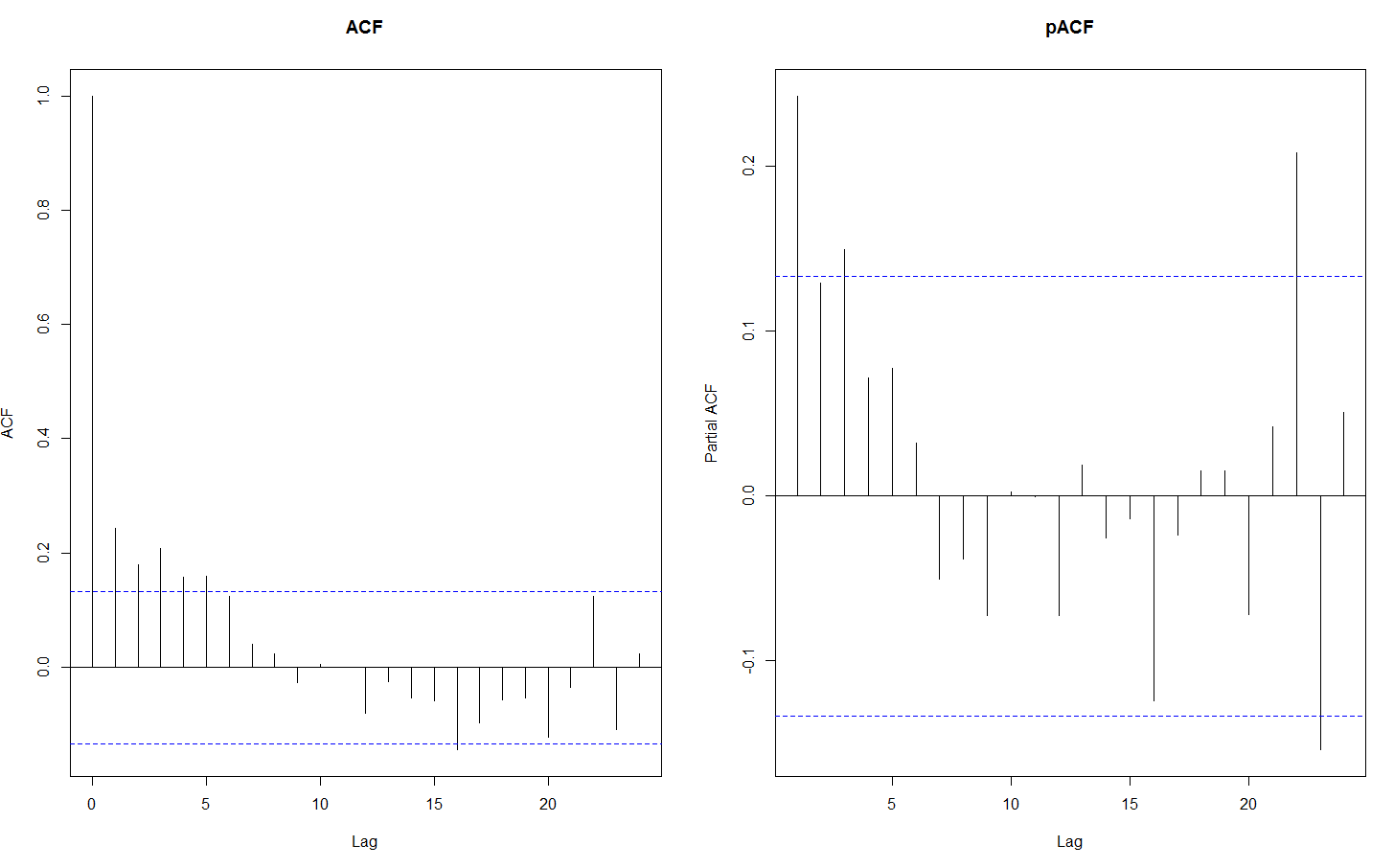


## **Supplementary Figure SF14.** ACF and pACF graphs to identify autocorrelation in mortality rate for ages 50-59.

##
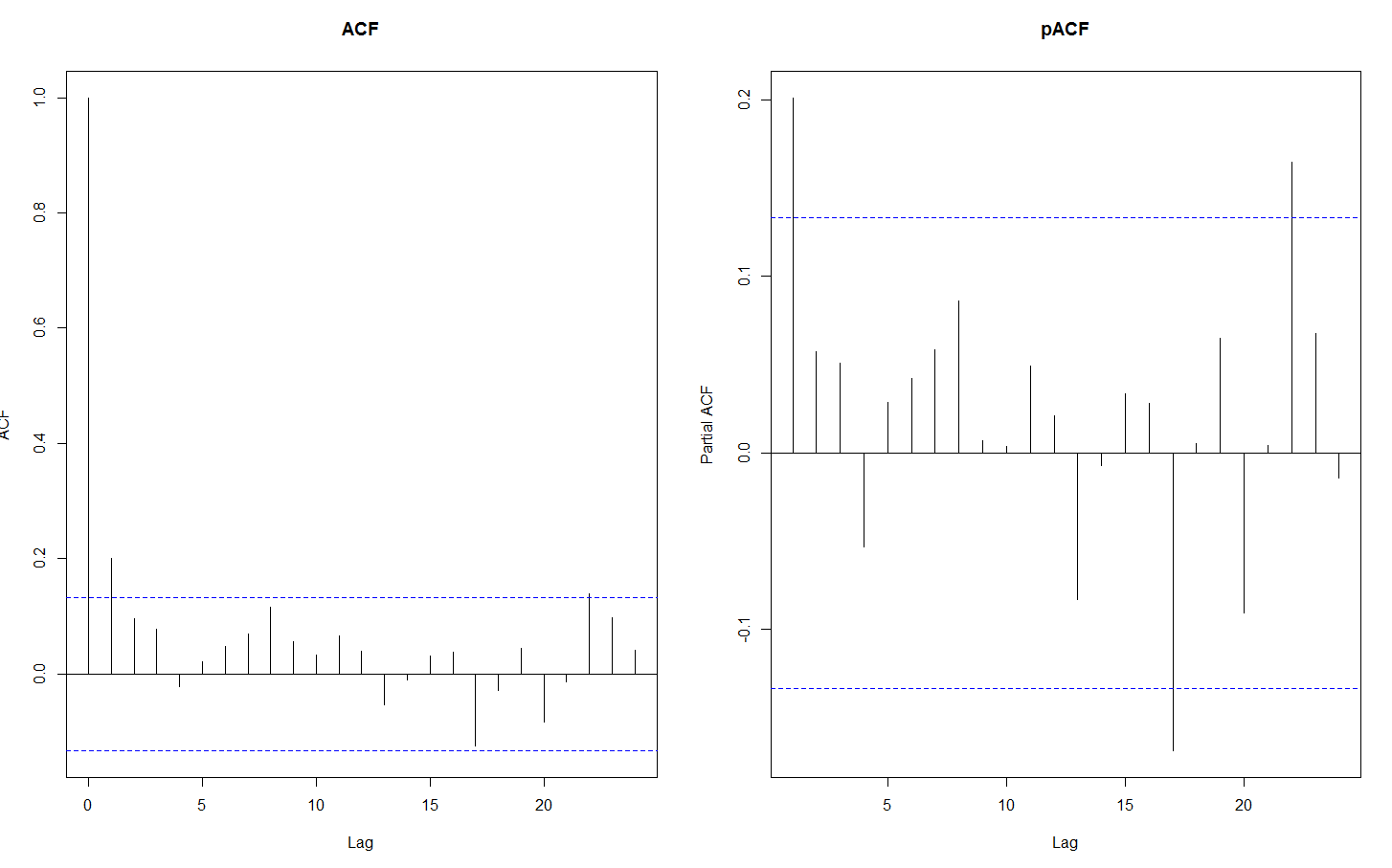
**Supplementary Figure SF15.** ACF and pACF graphs to identify autocorrelation in mortality rate for ages 60-69.


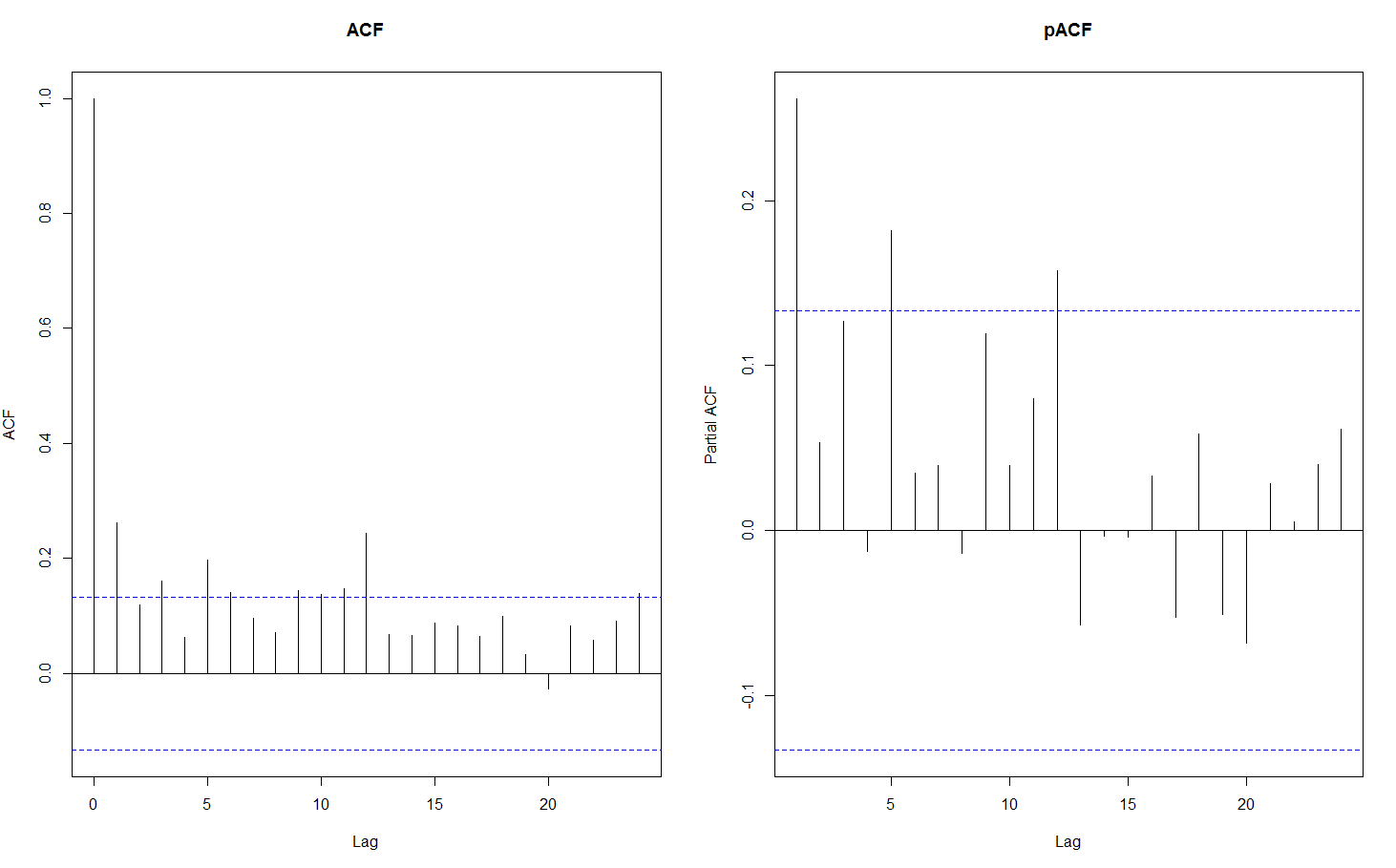


## **Supplementary Figure SF16.** ACF and pACF graphs to identify autocorrelation in mortality rate for ages 70-79.

##
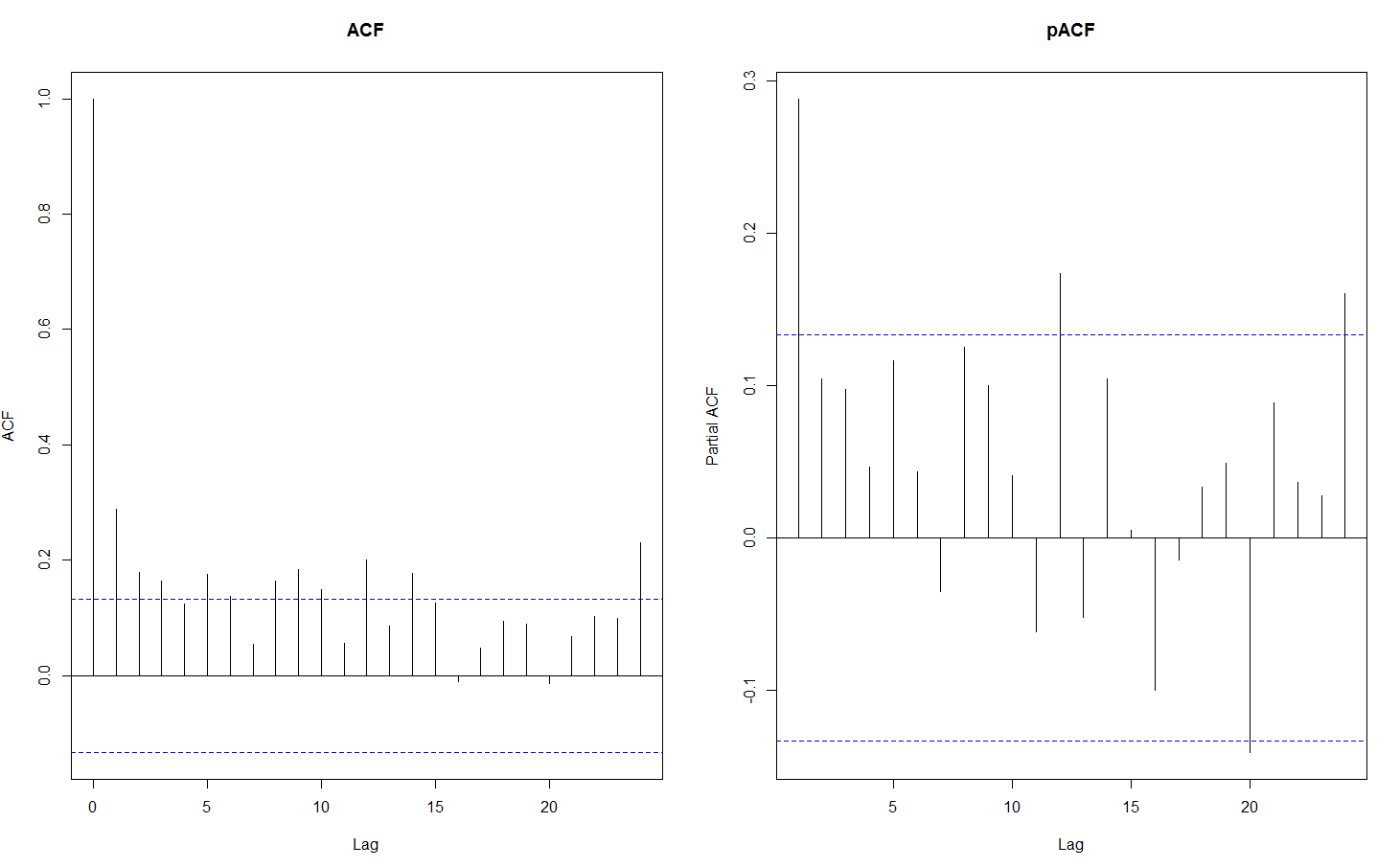
**Supplementary Figure SF17.** ACF and pACF graphs to identify autocorrelation in mortality rate for ages 80+.


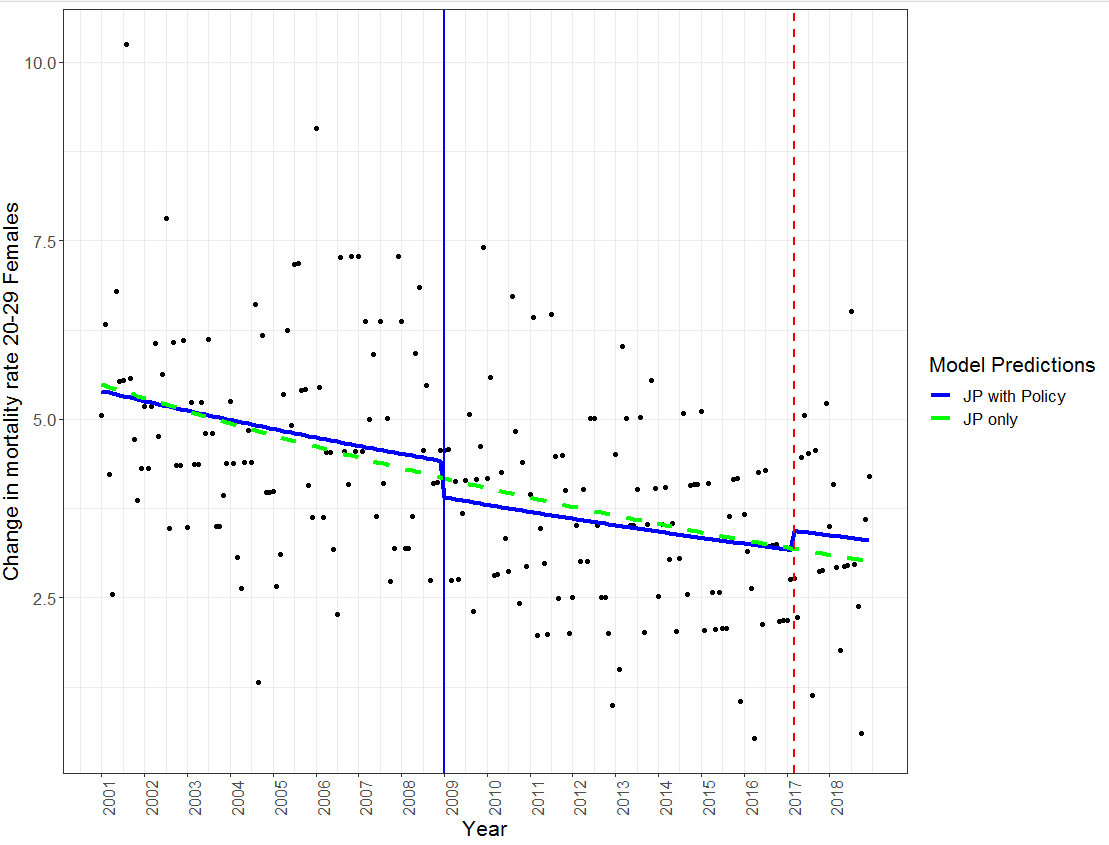

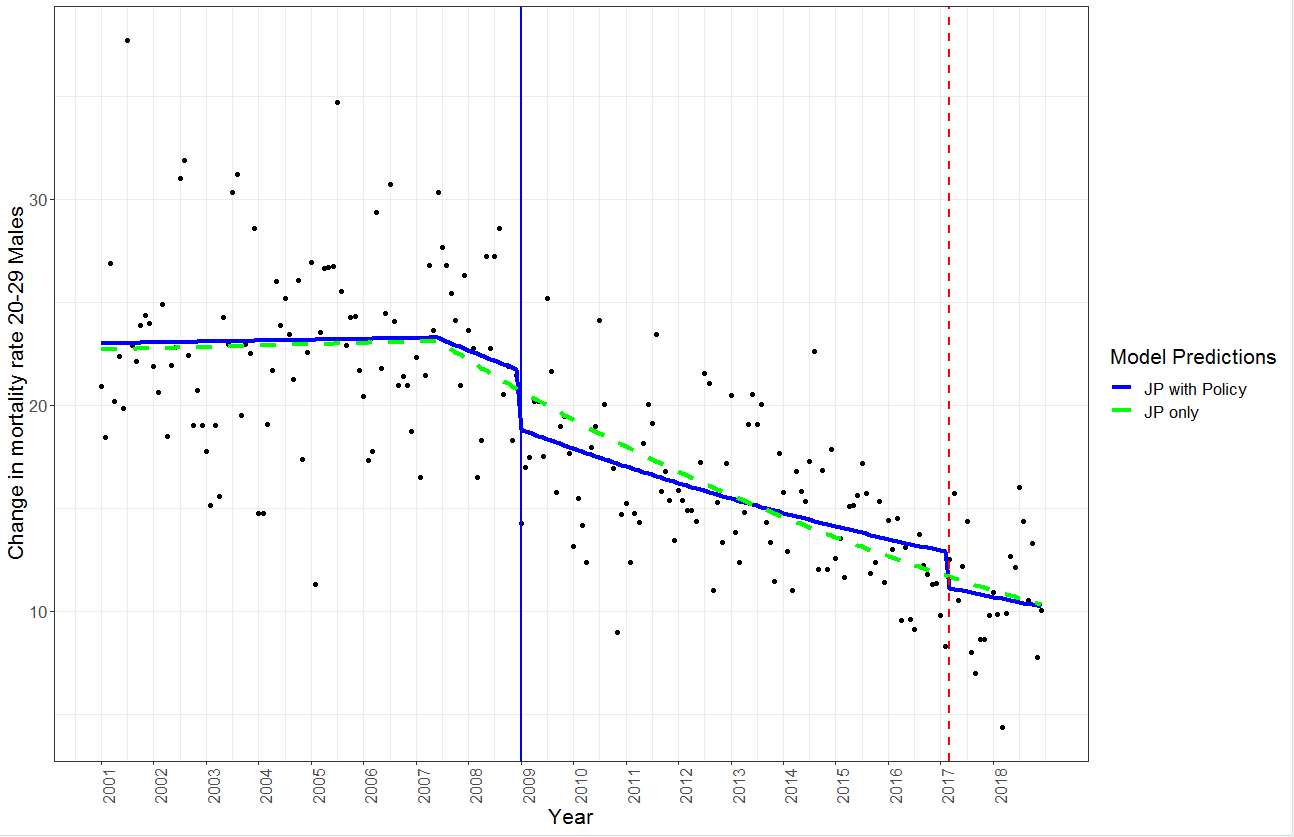


## **Supplementary Figure SF18.** Joinpoint analysis and Policy model for males and females ages 20-29.

##
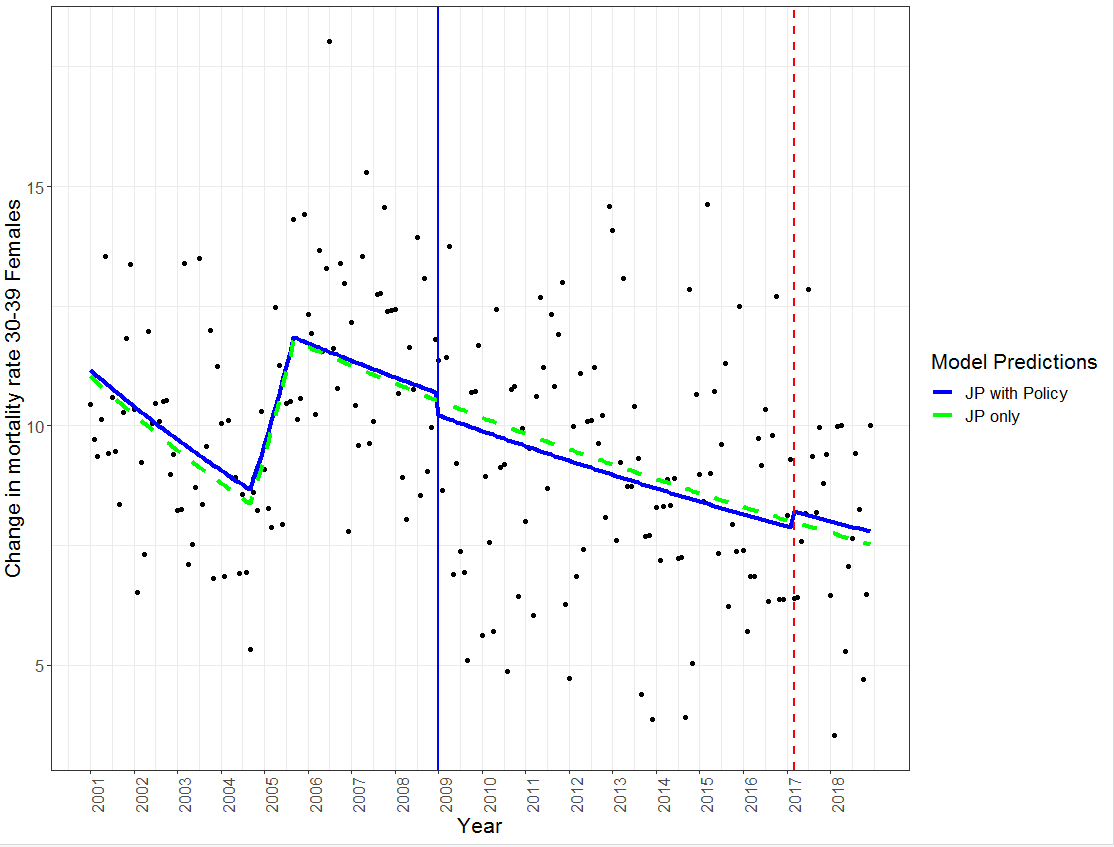

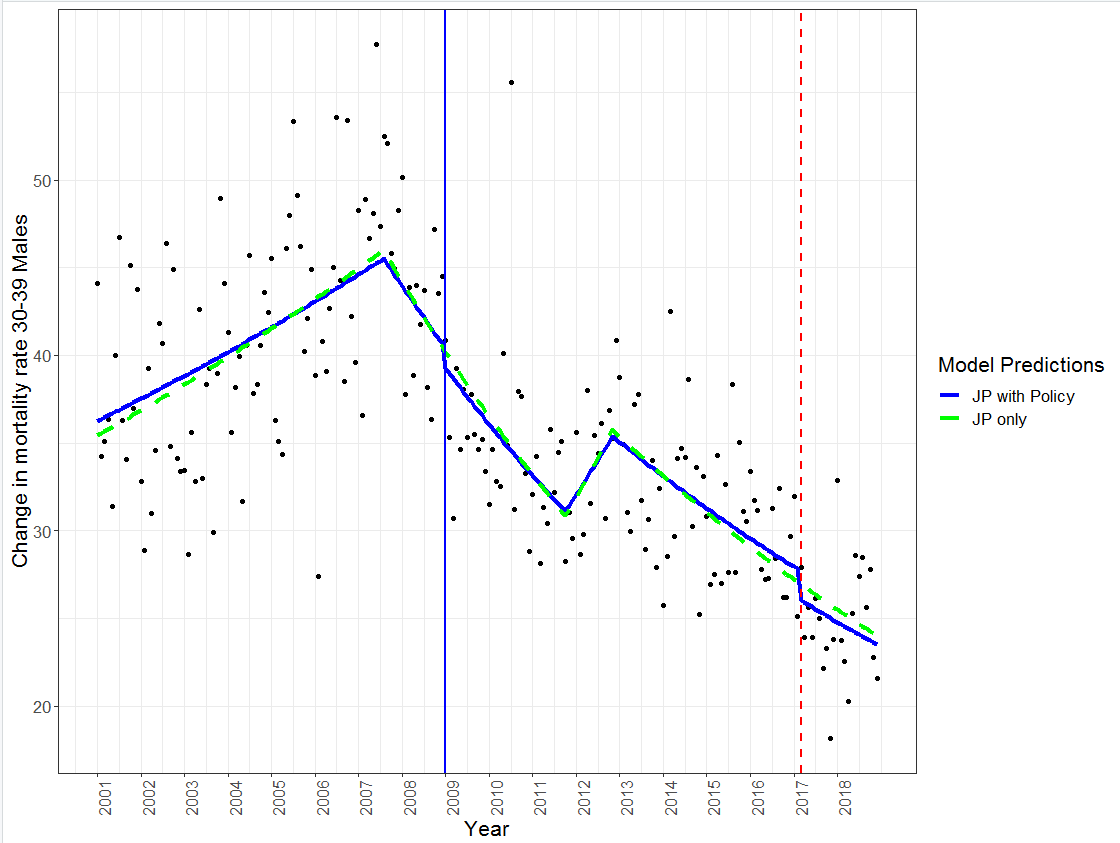
**Supplementary Figure SF19.** Joinpoint analysis and Policy model for males and females ages 30-39.

##
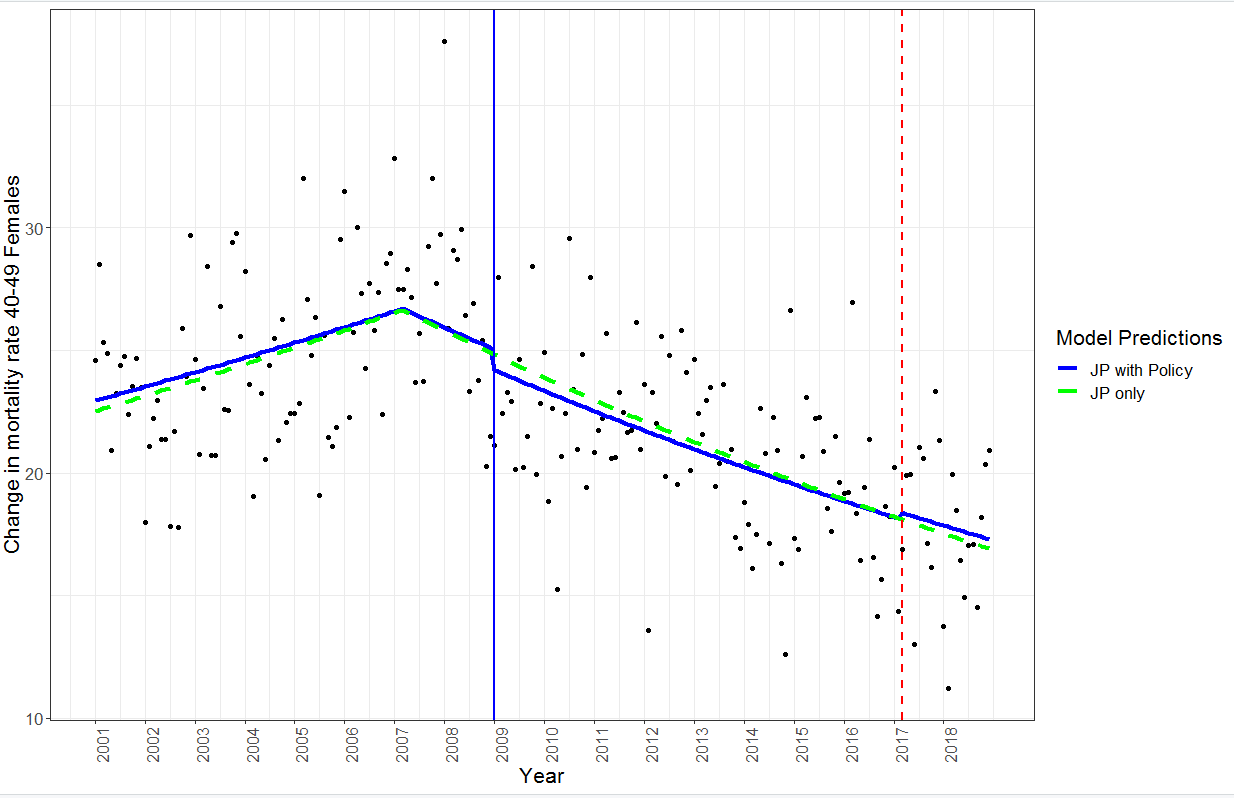

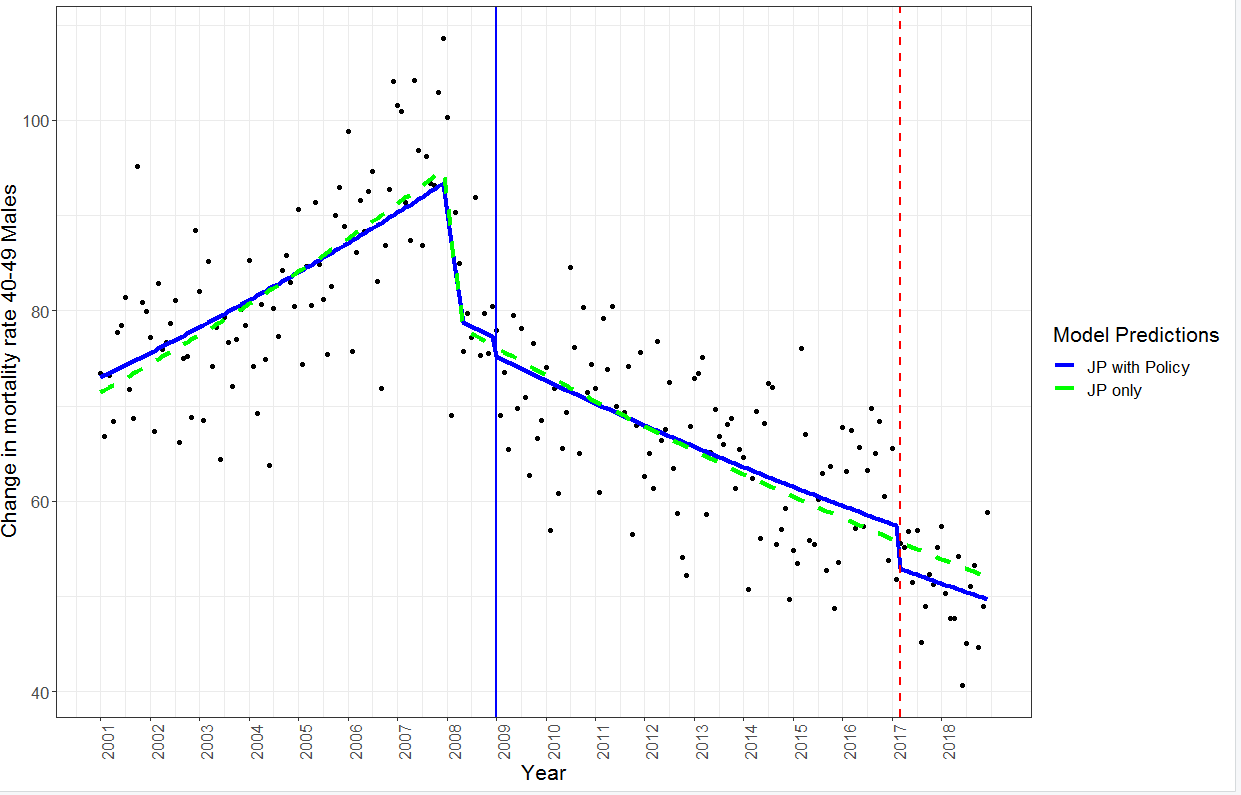
**Supplementary Figure SF20.** Joinpoint analysis and Policy model for males and females ages 40-49.

##
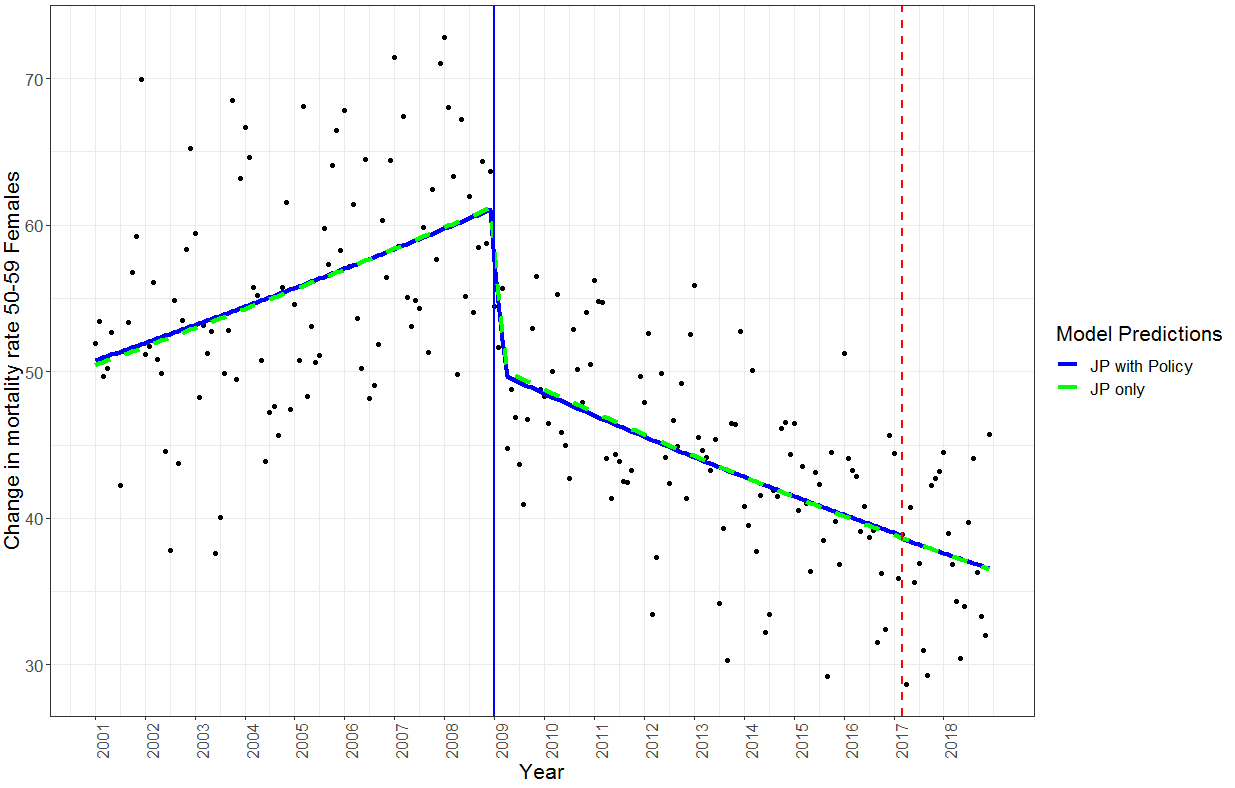

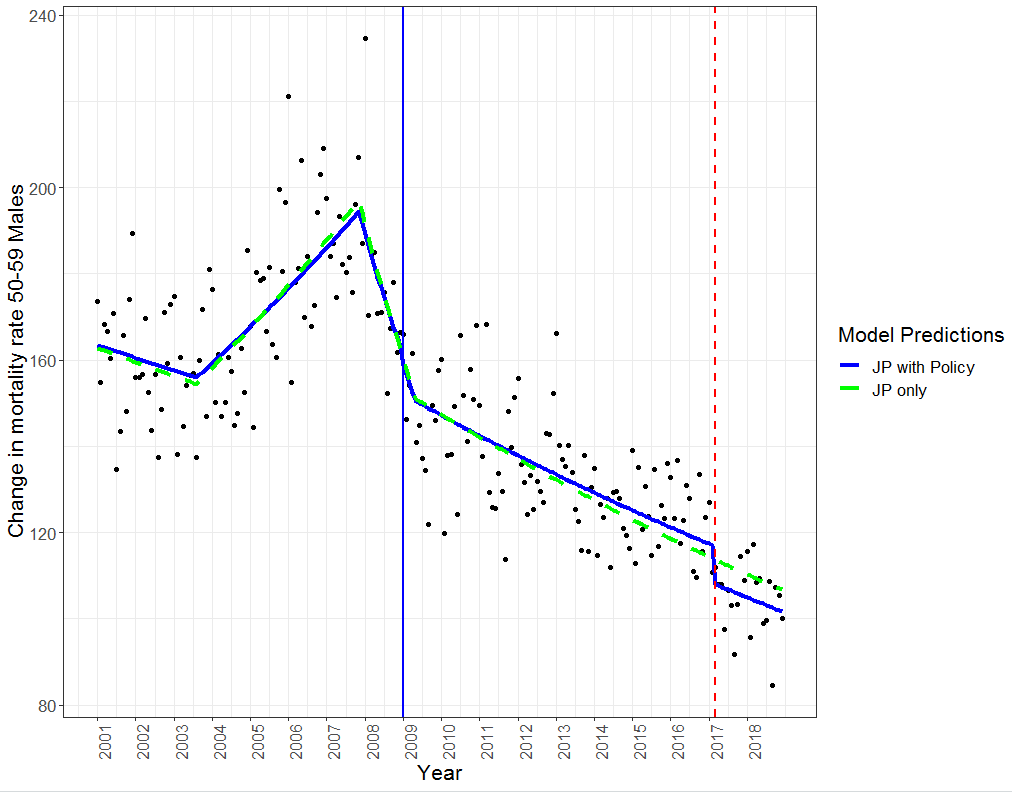
**Supplementary Figure SF21.** Joinpoint analysis and Policy model for males and females ages 50-59.

##
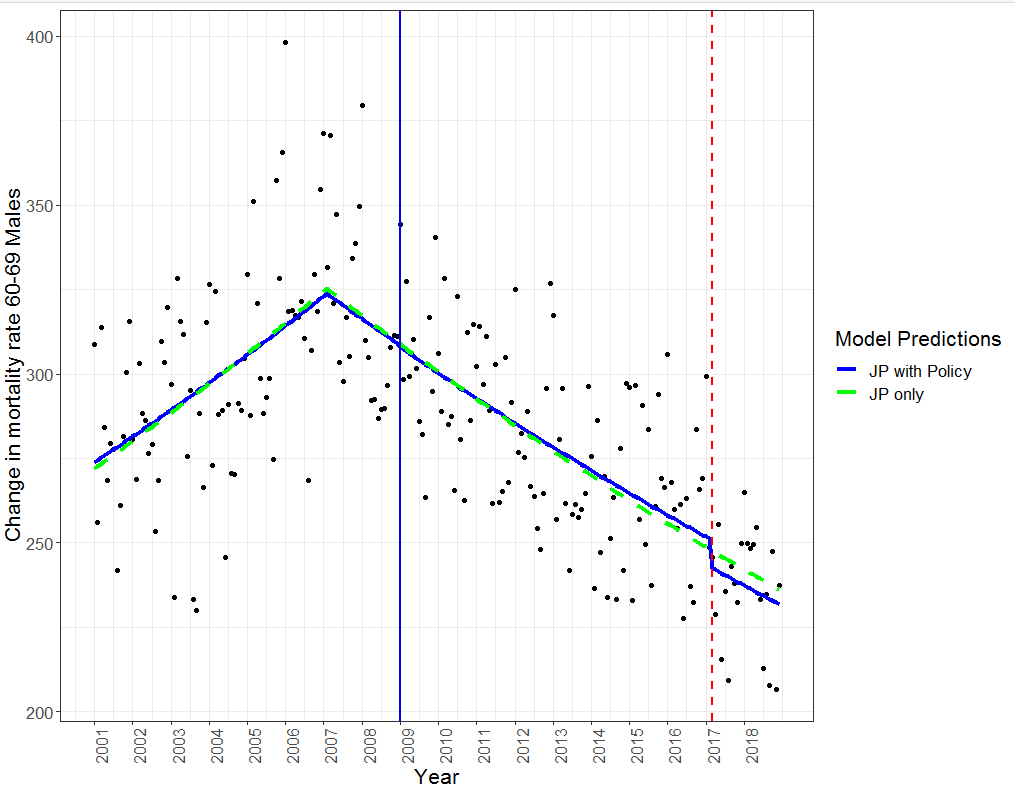

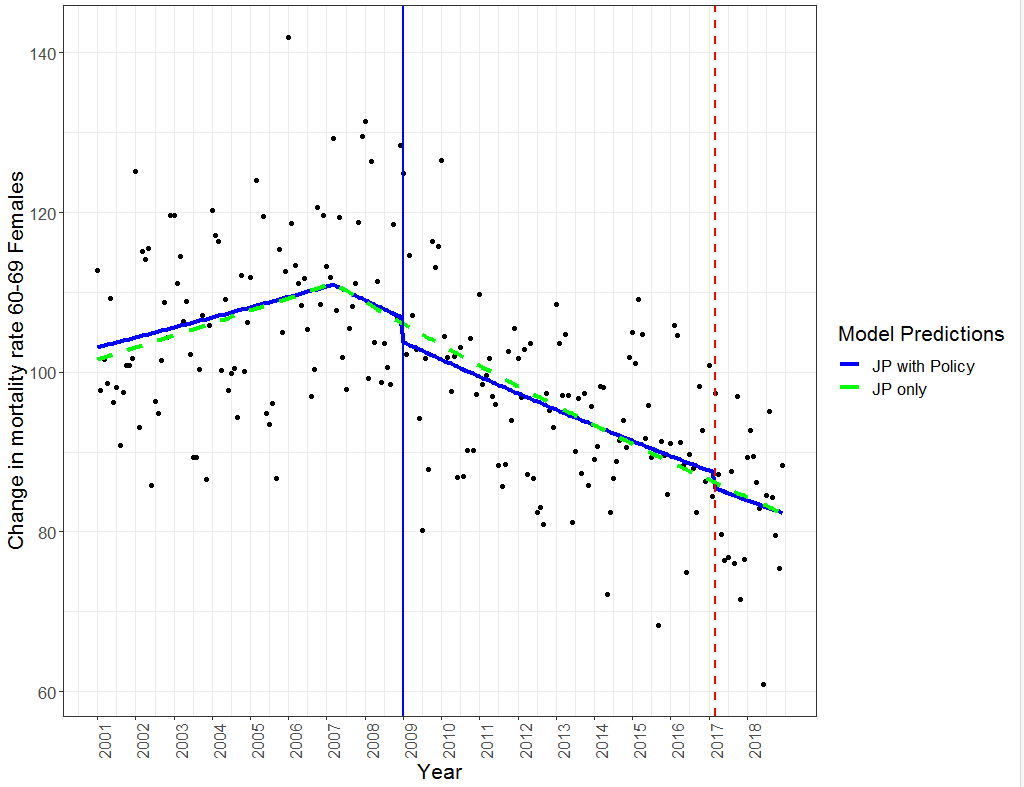
**Supplementary Figure SF22.** Joinpoint analysis and Policy model for males and females ages 60-69.

##
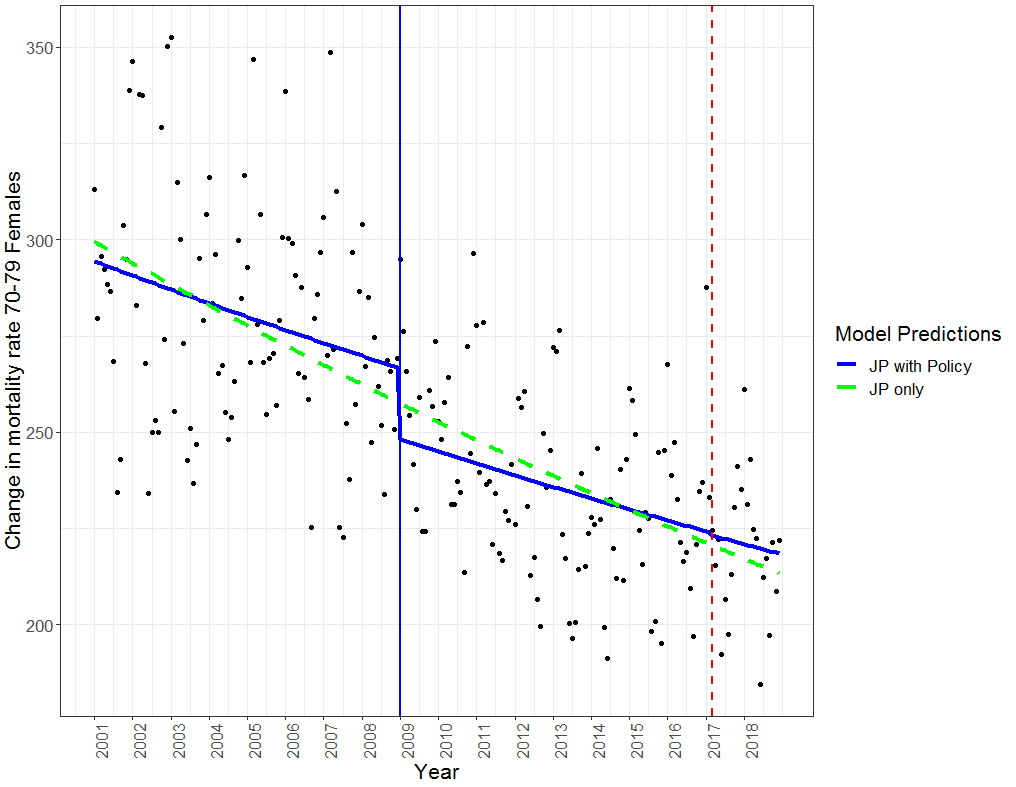

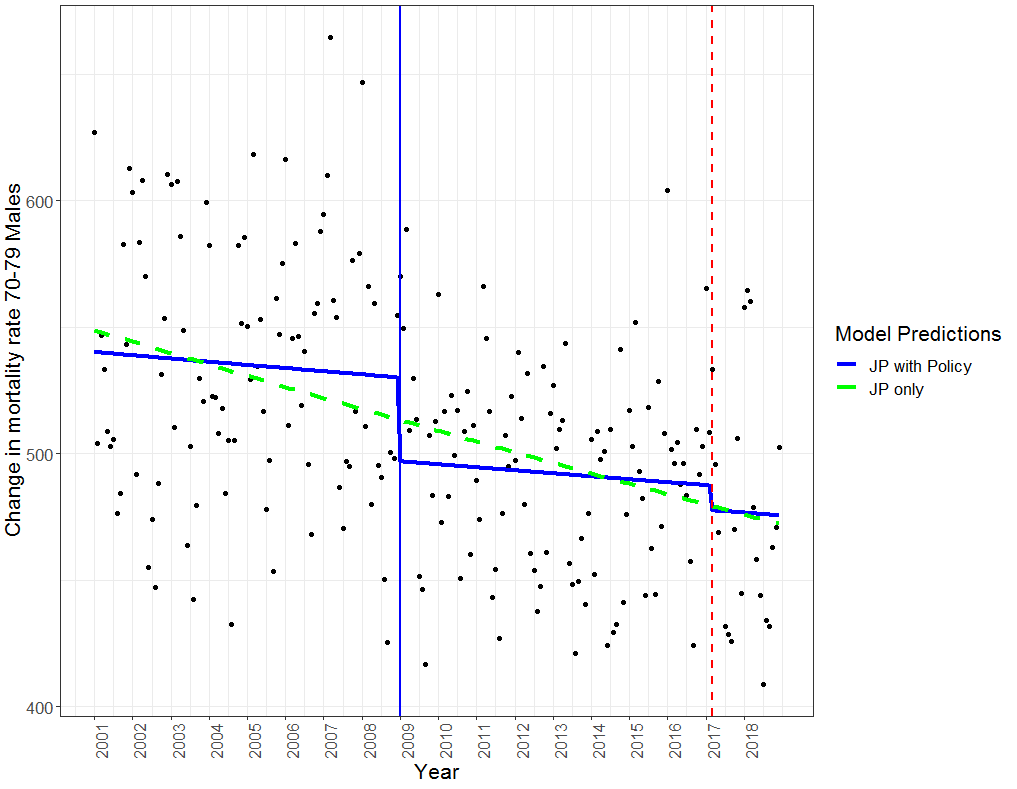
**Supplementary Figure SF23.** Joinpoint analysis and Policy model for males and females ages 70-79.

## **
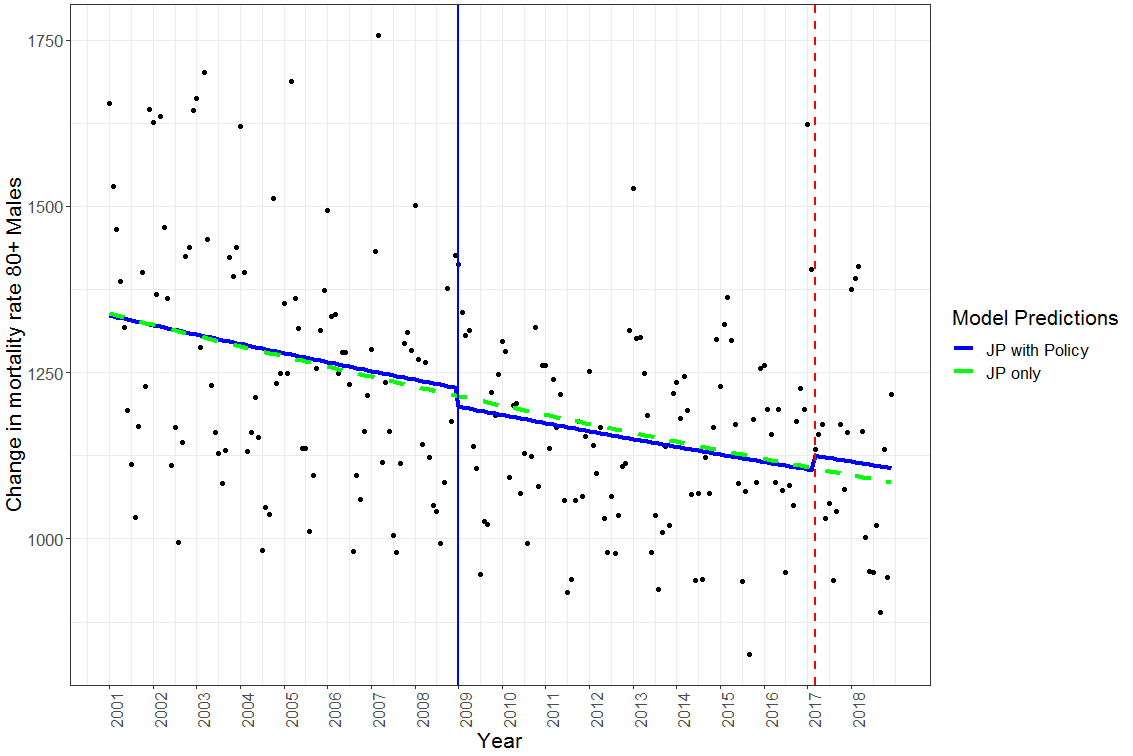

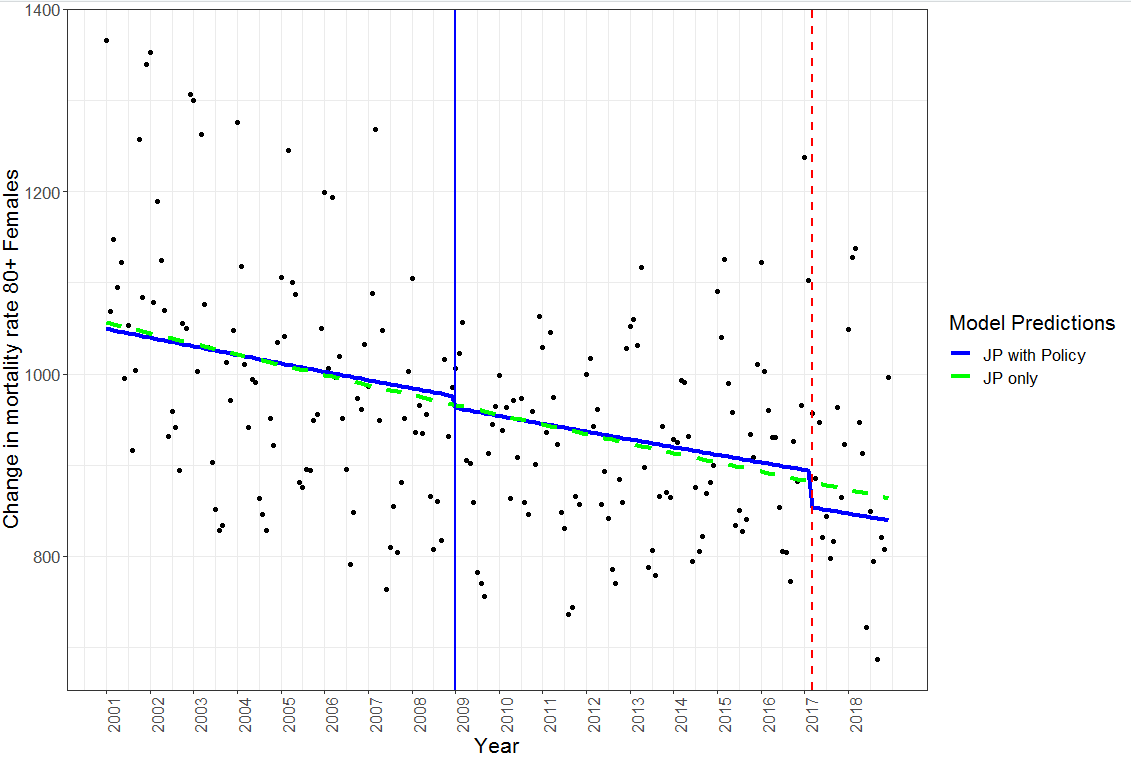
Supplementary Figure SF24.** Joinpoint analysis and Policy model for males and females ages 80+.
